# Longitudinal course of endocannabinoids and *N*-acylethanolamines in hair of mothers and their children in the first year postpartum: investigating the relevance of maternal childhood maltreatment experiences

**DOI:** 10.1017/S0033291723001204

**Published:** 2023-11

**Authors:** Melissa Hitzler, Lynn Matits, Anja M. Gumpp, Alexandra M. Bach, Ute Ziegenhain, Wei Gao, Iris-Tatjana Kolassa, Alexander Behnke

**Affiliations:** 1 Clinical and Biological Psychology, Institute of Psychology and Education, Ulm University, Ulm, Germany; 2 Division of Sports and Rehabilitation Medicine, Department of Medicine, Ulm University Hospital, Ulm, Germany; 3 Department of Child and Adolescent Psychiatry, Ulm University Hospital, Ulm, Germany; 4 Department of Biopsychology, Technische Universität Dresden, Dresden, Germany

**Keywords:** 2-/1-arachidonoylglycerol, anandamide, childhood maltreatment, endocannabinoids, intergenerational transmission, *N*-arachidonoylethanolamine, *N*-oleoylethanolamide, *N*-palmitoylethanolamide, *N*-stearoylethanolamide

## Abstract

**Background:**

Childhood maltreatment (CM) exerts long-lasting psychological and biological alterations in affected individuals and might also affect the endocannabinoid (eCB) system which modulates inflammation and the endocrine stress response. Here, we investigated the eCB system of women with and without CM and their infants using hair samples representing eCB levels accumulated during the last trimester of pregnancy and 10–12 months postpartum.

**Methods:**

CM exposure was assessed with the *Childhood Trauma Questionnaire*. At both timepoints, 3 cm hair strands were collected from mothers and children (*N* = 170 resp. 150) to measure anandamide (AEA), 2/1-arachidonoylglycerol (2-AG/1-AG), stearoylethanolamide (SEA), oleoylethanolamide (OEA), and palmitoylethanolamide (PEA).

**Results:**

Maternal hair levels of 2-AG/1-AG increased and SEA levels decreased from late pregnancy to one year postpartum. Maternal CM was associated with lower SEA levels in late pregnancy, but not one year later. In the children's hair, levels of 2-AG/1-AG increased while levels of SEA, OEA, and PEA decreased from late pregnancy to one year later. Maternal CM was not consistently associated with the eCB levels measured in children's hair.

**Conclusions:**

We provide first evidence for longitudinal change in the eCB system of mothers and infants from pregnancy to one year later. While maternal CM influenced the maternal eCB system, we found no consistent intergenerational effects on early regulation of the eCB system in children. Longitudinal research on the importance of the eCB system for the course and immunoregulation of pregnancy as well as for the children's development.

## Introduction

Adverse experiences during sensitive developmental periods cause profound and persistent psychological and physiological alterations that establish a lifetime vulnerability to negative sequelae of stress (Min, Minnes, Kim, & Singer, 2013; Nemeroff, 2016). Individuals with a history of childhood maltreatment (CM), i.e., sexual abuse as well as physical and emotional maltreatment and neglect, were shown to develop more mental and physical health problems than individuals without a CM history, especially when encountering additional stressors later in life (Hitzler et al., 2022; McLaughlin, Conron, Koenen, & Gilman, 2010; Thakkar & McCanne, 2000). Increased sensitivity to stress in CM-affected individuals was repeatedly linked to alterations in the regulation of physiological stress-response systems, including the hypothalamus-pituitary-adrenal (HPA) axis with its glucocorticoid hormone cortisol, as well as to increased reactivity of the immune system along with chronic low-grade inflammation (Boeck et al., 2016; Carpenter et al., 2007; Danese & Baldwin, 2017; Koenig et al., 2018a; Strueber, Strueber, & Roth, 2014). However, findings on HPA-axis activity and inflammation in the context of CM are still incomplete and their interplay with other biological mechanisms is insufficiently understood.

Therefore, recent research on the consequences of early adversity has increasingly focused on the endocannabinoid (eCB) system (e.g. Bassir Nia, Bender, & Harpaz-Rotem, 2019; Behnke et al., 2020; Koenig et al., 2018b), as it critically co-regulates the HPA axis and the activity of immune cells (review in Hauer, Toth, & Schelling, 2020; Hillard, 2018; Riebe & Wotjak, 2011). The eCB system comprises the eCBs anandamide (*N*-arachidonoylethanolamine, AEA) and 2-arachidonoylglycerol (2-AG), their endogenous cannabinoid receptors (e.g. CB_1_ and CB_2_), and degrading enzymes such as fatty acid amide hydrolase (FAAH) (Joshi & Onaivi, 2019). eCB are bioactive signaling lipids that allow fast adaption to changing stress conditions as they are synthesized on demand from cell membranes of the central nervous system, blood and immune cells, and other peripheral tissues (Tsuboi, Uyama, Okamoto, & Ueda, 2018). Furthermore, the eCB system comprises various *N*-acylethanolamides (NAEs), including palmitoylethanolamide (PEA), oleoylethanolamide (OEA), and stearoylethanolamide (SEA) (Dlugos, Childs, Stuhr, Hillard, & de Wit, 2012), which are structurally highly similar to AEA and may enhance its effects (Ho, Barrett, & Randall, 2008; Jonsson, Vandevoorde, Lambert, Tiger, & Fowler, 2001). NAEs do not seem to be biologically active under physiological conditions, but rather exhibit their properties and functions only under certain conditions, i.e., stress (Hauer et al., 2013; 2020). The eCB system acts as a pivotal co-regulator of HPA-axis activity: AEA suppresses HPA-axis activity by binding to CB_1_ receptors (Hill et al., 2011). Confronted with a stressor, FAAH rapidly degrades AEA to disinhibit the HPA axis which eventually initiates the secretion of glucocorticoids from the adrenal glands. As negative feedback, the secretion of cortisol stimulates the synthesis of 2-AG which restores HPA-axis homeostasis and normalizes AEA levels (Bassir Nia et al., 2019; Hauer et al., 2020). This regulation is also mirrored through negative associations between 2-AG and glucocorticoid levels measured in the hair of adults (Behnke et al., 2021; 2020) and during pregnancy (Krumbholz, Anielski, Reisch, Schelling, & Thieme, 2013).

Moreover, eCBs, NAEs, and their receptors seem to modulate inflammatory activity by downregulating inflammation and pain via different pathways (Berdyshev et al., 2015; Dalle Carbonare et al., 2008; Gallego-Landin, García-Baos, Castro-Zavala, & Valverde, 2021; Hillard, 2018). For example, to restore immune reactions back to baseline, 2-AG binds to CB_2_ receptors on immune cells, reducing their release of pro-inflammatory cytokines (Hillard, 2018; Tsuboi et al., 2018). SEA, PEA, and OEA were also found to reduce peripheral inflammation and cytokine secretion (Berdyshev et al., 2015; Dalle Carbonare et al., 2008). Correspondingly, a number of studies linked higher peripheral eCB and NAE concentrations to inflammatory states (Barrie & Manolios, 2017; Berdyshev et al., 2015; Crowe, Nass, Gabella, & Kinsey, 2014). Despite these results, the regulatory direction in the crosstalk of eCBs and NAEs and the immune response is not yet fully understood and could also be bidirectional (Hauer et al., 2020).

As the eCB system modulates endocrine stress and immune homeostasis, research has highlighted the importance of the eCB system in the etiology of stress-related mental health problems. Although this is supported by studies showing reduced blood levels of eCBs and NAEs in individuals with PTSD (Hill et al., 2013; Neumeister, Seidel, Ragen, & Pietrzak, 2015) and major depression (Hill, Miller, Ho, Gorzalka, & Hillard, 2008; 2009), other studies have failed toreplicate these associations or have even found the contrary (Behnke et al., 2021; deRoon-Cassini et al., 2022; Hauer et al., 2013; Romero-Sanchiz et al., 2019). While circulating eCB concentrations in blood are highly fluctuating depending on circadian rhythmicity and acute stressors (Vaughn et al., 2010; Voegel, Baumgartner, Kraemer, Wüst, & Binz, 2021), hair analyses provide a more stable retrospective measurement of long-term eCB accumulation over weeks and months (Gao, Schmidt, Enge, & Kirschbaum, 2021; Krumbholz et al., 2013). In hair, higher eCB and NAE levels were associated with depressive symptoms and CM (Behnke et al., 2020; Croissant et al., 2020), whereas trauma-exposed individuals with and without PTSD showed reduced levels of eCBs and NAEs in hair (Wilker et al., 2016).

Previously, we provided initial evidence on the relevance of the eCB system for the intergenerational transmission of CM: Using hair samples to represent the last trimester of pregnancy, we found that mothers with a CM history showed higher hair concentrations of 1-AG and of lower SEA compared to mothers without CM. Correspondingly, their newborns showed higher levels of 1-AG and OEA as compared to newborns of mothers without a CM history (Koenig et al., 2018b). CM-related alterations in newborn eCB and NAE levels could indicate that children are intergenerationally affected by the consequences of their mothers' CM experiences. However, it is unclear to date, whether these CM-related alterations in eCB and NAE levels persist in mothers and their children beyond the physiologically challenging period of pregnancy and birth. In general, little is known about the temporal fluctuation of eCBs in hair. First results indicate a relatively low intraindividual variation in healthy adults (Gao et al., 2021); however, eCB levels were reported to fluctuate across pregnancy (Krumbholz et al., 2013). It is to be investigated whether the postpartum period involves alterations in the eCB system. This is quite conceivable, since successful pregnancy depends on the time- and tissue-specific regulation of eCBs and NAEs within the reproductive system (Fonseca et al., 2010a, 2010b; Kozakiewicz, Grotegut, & Howlett, 2021; Maia, Fonseca, Teixeira, & Correia-da-Silva, 2020; Taylor et al., 2010), and since extensive (ovarian) hormonal and immunological transitions from pregnancy to the postpartum recovery presumably affect the eCB system (Kozakiewicz et al., 2021; Lam et al., 2008). Likewise, the early development of the eCB system in newborns has not yet been characterized.

Therefore, this study is the first to characterize the longitudinal development of the eCB system in mothers and newborns during the first year postpartum. Building on our previous findings (Koenig et al., 2018b), we expected altered maternal and infant eCB and NAE hair concentrations in the last trimester of pregnancy depending on maternal CM history. With the present study, we provide novel evidence on how intergenerational CM-related alterations in the eCB system evolve in the first year postpartum.

## Materials and methods

### Participants and study procedures

Female participants were recruited for the longitudinal study ‘My Childhood – Your Childhood’ which investigates risk and resilience factors in the intergenerational transmission of CM in a healthy community sample of mother–infant dyads (for details see Hitzler et al., 2022 and online Supplementary Fig. S1). Women were approached in the maternity ward shortly after parturition [*t*_0_: on average after *M* (s.d.) = 2.6 (1.7) days] and for follow-up measurements at 3 months (*t*_1_) and 12 months (*t*_2_) after birth. Exclusion criteria for study participation were insufficient knowledge of German language, severe complications during parturition (e.g. stillbirth), severe health problems of mother or child (e.g. admission to intensive care), and maternal age under 18 years. Online Supplementary Figs S1 and S2 detail study flow, recruitment process, and dropout rates of all measurement points. All study procedures have been approved by the Ulm University ethics committee and were in accordance with the Declaration of Helsinki.

Hair was collected from mothers (*t*_0_: *N* = 474; *t*_2_: *N* = 244) and children (*t*_0_: *N* = 331; *t*_2_: *N* = 237) at *t*_0_ and *t*_2_. Due to limited material, the analysis of steroid hormones was prioritized (data not shown here). At *t*_0_, sufficient material for additional eCB and NAE quantification was available for 150 mothers and 92 children; and at *t*_2_, eCB and NAE levels were measured in the hair of 148 mothers and 170 children. Complete eCB and NAE data from both measurement points were available from in a subsample of *N* = 63 mothers and *N* = 45 children (see online Supplementary Fig. S2 for details).

Sociodemographic, clinical, and hair characteristics of the investigated sample can be found in Table 1. Except for a higher severity of reported CM experiences, mothers with and without CM showed no statistically significant differences in any of the descriptive characteristics. Children with or without maternal CM did not differ in any relevant descriptive characteristics.

**Table 1.** Sociodemographic and clinical characteristics for mothers and children shortly after parturition (*t*_0_) and 12 months postpartum (*t*_2_)

	Mothers	
	*t*_0_, *N* = 150	*t*_2_, *N* = 148
Sociodemographics		
Age in years (*M,* s.d.)	32.60 (4.72)	34.32 (4.33)
German origin (*n, %*)^a^	129 (86)	127 (85.80)
Higher education (*n, %*)*	129 (86)	141 (93.20)
Living in committed relationship (*n, %*)	146 (97.30)	146 (98.60)
Number of children (*M,* s.d.)	1.60 (1.09)	1.62 (0.80)
Smoking (*n, %*)	12 (8)	12 (8.10)^b^
Gestation in days (*M,* s.d.)	275.25 (12.30)	277 (9.25)
Cannabis intake (*n*, %)	0	0
BMI (kg/m^2^) (*M,* s.d.)^c^	–	24.84 (5.15)^d^
Psychological & Clinical characteristics		
Psychiatric disorder (*n*, %)^e^		
Lifetime	33 (22)	58 (39.20)^d^
Current	–	7 (4.70)^f^
Psychotropic medication lifetime (*n,* %)	33 (22.0)^g^	30 (20.30)^f^
Chronic somatic illness (*n*, %)	53 (35.60)^g^	54 (36.50)^h^
Psychotherapy/ counseling, lifetime (*n*, *%*)	57 (38)	66 (44.60)^†^
Medication intake (*n*, %)	72 (48)^g^	67 (45.30)^b^
Childhood maltreatment (CTQ; *M,* s.d.)	35.02 (13.54)	34.57 (11.71)
Emotional abuse (*M,* s.d.)	7.37 (4.04)	7.35 (3.70)
Physical abuse (*M,* s.d.)	6.16 (2.89)	5.93 (2.53)
Sexual abuse (*M,* s.d.)	6.31 (3.95)	5.99 (3.20)
Emotional neglect (*M,* s.d.)	9.22 (4.45)	9.48 (4.43)
Physical neglect (*M,* s.d.)	5.96 (1.93)	5.87 (1.75)
Hair related characteristics		
Weekly hair washing frequency (*M,* s.d.)	3.48 (1.72)	3.44 (1.70)^i^
Hair treatment (*n,* %)^‡^	101 (67.30)	72 (48.60)^f^
Natural hair color^j^		
Blonde/red	68 (45.30)	72 (49)
Brunet/black	79 (52.70)^g^	71 (48.30)^b^
	Children	
	***t*_0_**, *N* = 92	***t*_2_**, *N* = 170
Female sex (*n, %*)	42 (45.70)	79 (46.50)
Weight in kg (*M,* s.d.)	3.44 (0.48)	9.66 (1.08)
Age in days (*M,* s.d.)	2.3 (1.66)	381.5 (32.68)
Regular Medication since birth (*n, %*)	–	28 (16.50)^k^
Intake acute medication (*n, %*)	–	15 (8.80)
Current illness (*n, %*)	–	20 (11.80)^k^
Serious/chronic illness since birth (*n, %*)	–	15 (8.80)^k^
Natural hair color (*n,* %)		
Blonde/red	6 (6.50)	14 (8.20)
Brunet/black	86 (93.50)	106 (62.40)^l^

*Note*: Sample characteristics were calculated with the maximal number of cases available: ^b^ *N* = 143; ^d^ *N* = 140; ^f^ *N* = 142; ^g^ *N* = 149; ^h^ *N* = 146; ^i^ *N* = 96; ^k^ *N* = 164; ^l^ *N* = 120.

a Other origins in descending order: Eastern Europe; Brasil, Austria, France; Africa.

c BMI was not calculated at t_0_, as body weight was not reliable due to weight changes in pregnancy.

e Mainly major depressive and anxiety disorders.

j Only a small percentage of mothers had red (*n* = 4) or black (*n* = 3) as natural hair color.

* At least 10 years of high school education.

† *n* = 9 women started counseling since birth.

‡ Hair treatment: Coloration, permanent waving; regular use of curling iron or hair straightener; hair bleaching; other chemical hair treatment.

### Clinical measures

After obtaining written informed consent, socio-demographic, clinical, and hair characteristics (hair treatment: coloration, bleaching, waving; frequency of hair washing) were assessed in a diagnostic interview (t_0_; see Table 1). In addition, hair samples of mothers and newborns were collected. A maternal history of CM was retrospectively assessed using the German version of the *Childhood Trauma Questionnaire* (CTQ; Bader, Hänny, Schäfer, Neuckel, & Kuhl, 2009; Bernstein & Fink, 1998) covering the five subscales emotional, physical, and sexual abuse, as well as emotional and physical neglect. Due to the emotionally sensitive situation of the participating women, trained psychologists conducted the CTQ as an interview to ensure adequate care in case of possible psychological distress during the recording of stressors. The cumulative severity of CM experiences (CM load) was operationalized through the CTQ sum score (possible range: 25–125). Around 12 months postpartum [*t*_2_: *M* (s.d.) = 378.0 (34.9) days after birth], the women were re-invited to a second follow-up, comprising a psychological interview as well as the assessment of clinical, medical, and hair-related data (see Table 1).

### Hair endocannabinoid analysis

#### Hair collection

At *t*_0_ and *t*_2_, hair samples were collected and processed by trained academic staff, using laboratory gloves to avoid contamination of hair with skin moisture. In mothers, optimally three hair strands (~3 mm diameter each) were cut close to the scalp from the posterior vertex position. If this sampling location was not possible in the children due to sparse hair, samples were taken from the sites where the most hair was present, usually at the hairline beneath the ear. The newborns' hair collected after parturition (*t*_0_) was washed with clear water to preclude contamination with blood or amniotic fluid.

#### Pre-processing

In a standardized procedure, the 3 cm hair segment was cut proximal to the scalp. Due to an approximate adult hair growth of ~1 cm/month (Wennig, 2000), the 3 cm hair segment proximal to the scalp reflects maternal cumulative eCB concentration incorporated in the last trimester of pregnancy. Fetal/neonatal hair grows slower with ~1 cm in three months during the whole third trimester of pregnancy (cf. Gareri and Koren, 2010). Therefore, to display the metabolic activity during the last three prenatal months, hair from newborns collected at *t*_0_ was cut into 1 cm segments. At *t*_2_, the proximal 3 cm hair segment of mothers and children was used for analyses, reflecting month 10 to 12 postpartum (for details see online Supplementary information S1). Cut hair was weighed (range 4–6 mg) and placed into Falcon tubes. For sample details and missing data see online Supplementary information S1 and Fig. S2.

#### Mass spectrometric measure of eCB and NAE

The hair eCB concentrations of AEA, 2-AG, OEA, SEA, and PEA were quantified with LC-MS/MS mass spectrometry following the previously published protocol of Gao, Walther, Wekenborg, Penz, and Kirschbaum (2020). At *t*_0_, AEA quantification was only successful in a small subsample of mothers. Moreover, as AEA concentrations are rather low in hair, AEA in the current cohort had most values under detection limit, even when sufficient material was available for analysis (see online Supplementary Fig. S2). Thus, AEA had to be precluded from some of the subsequent analyses. Note that the measure of 2-AG is combined with its biologically inactive analog 1-AG that is rapidly isomerized from 2-AG through an acyl-group migration (Sugiura et al., 1996), presumably due to the extraction method during the analyses process (Zoerner et al., 2012). Hence, a commonly used approach is to sum the acquired individual peak areas of 2-AG and 1-AG, assuming that 1-AG originates primarily from 2-AG. Thus, the combined measure of 2-AG and 1-AG is indicated as 2-AG/1-AG in this study.

### Statistical analyses

Statistics were calculated with R version 4.2.1 (R Core Team, 2019). In case of non-normal distributed or non-interval scaled variables, Spearman's rank correlations (*r*_s_) were computed. Depending on normality and equality of variances, groups were compared using independent Student's *t* tests and non-paired Wilcoxon rank-sum tests.

Linear mixed-effects models were calculated to assess how eCB and NAE hair concentrations evolve in the first year after birth depending on CM. As the model assumptions (i.e. normality of model residuals) were not met, robust linear mixed-effects models were conducted using the *robustlmm* package (Koller, 2016). The models predicted the eCB and NAE levels by the fixed within factor ‘Time’ (*t*_0_ *v. t*_2_) and the fixed between factor ‘Maternal CM load’ along with the interaction Time × Maternal CM load. To reflect the repeated measures, we modeled intercepts for each subject as a random effect (Blackwell, De Leon, & Miller, 2006). The robust mixed-effects regression models did not allow calculating overall model statistics. The nature of significant Time × Maternal CM load interactions was explored using *post hoc* tests (i.e. linear contrasts) using the *emmeans* package (Lenth, 2019). *P* values of model predictors and *post hoc* tests were calculated based on *z* values.

All reported analyses were performed two-tailed with the significance level set at *p* < 0.05. *P* values of the bivariate correlations between CM exposure and the biological measures as well as for *post hoc* tests and descriptive analyses were adjusted using the False Discovery Rate (FDR) (Benjamini & Hochberg, 1995).

Relevant covariates did not significantly correlate with eCB and NAE levels (see online Supplementary Tables S5 & S6). Hair washing reported by mothers and the children's sex did not correlate with eCBs and NAEs. Considering frequency of hair washing and infant sex as covariates in sensitivity analyses did not change the pattern of the results.

## Results

### Descriptive and correlational results

The online Supplementary Tables S1 & S2 present a summary of the eCB and NAE concentrations measured in the hair of mothers and their newborns. In line with previous studies (Gao et al., 2020; Koenig et al., 2018b), eCB and NAE concentrations in maternal and infant hair showed a comparably wide physiological range. On average, the eCB and NAE levels were higher in mothers than in children at *t*_0_ and *t*_2_. Exceptionally, hair of newborns collected at birth exhibited, on average, three times higher 2-AG/1-AG levels than maternal hair after giving birth. PEA, OEA, and SEA concentrations were all positively correlated in both mothers and children at each time of measurement (*r*_S_ = 0.31–0.93, all *p_FDR_* < 0.001; see online Supplementary Table S3). There was no consistent pattern of bivariate associations between AEA, 2-AG/1-AG, and the NAE in mothers or children at any point in time (see online Supplementary Table S3).

Within mother–infant dyads, maternal and infant eCB and NAE levels did not show significant intergenerational correlation at any point in time (all *p_FDR_* > 0.05; online Supplementary Table S3). As an exception, there was a significant negative correlation of maternal SEA and infant 2-AG/1-AG (*r_S_* = −0.44, *p_FDR_* < 0.001) in hair collected at 12 months postpartum.

Regarding within-subject correlations, maternal OEA, SEA, and PEA hair levels were positively correlated between *t*_0_ and *t*_2_. In contrast, there were no associations of children's eCB and NAE between *t*_0_ and *t*_2_ (see online Supplementary Table S4).

### Association of maternal CM history with eCB and NAE hair concentrations in the first year postpartum^†^^1^

#### Maternal hair

Table 2 displays the results of bivariate Spearman correlation analyses in mothers. During the last trimester of pregnancy, mothers with higher CM load showed significantly lower SEA hair concentrations and in trend higher 2-AG/1-AG. In addition, when exploring associations of CM subtypes with the biological measures (online Supplementary Table S5), we found that women with higher emotional abuse showed lower SEA levels at one year postpartum. However, the correlation was not significant after FDR correction.

**Table 2.** Spearman rank correlations of maternal childhood maltreatment exposure with endocannabinoids measured in maternal hair and infant hair

		Hair samples collected shortly after parturition (*t*_0_) representing the last trimester of pregnancy					Hair samples collected 12 months postpartum (*t*_2_) representing 10–12 months postpartum				
		Mothers, *N* = 150					Mothers, *N* = 148				
		AEA^a^	2-AG/1-AG	OEA	SEA	PEA	AEA^b^	2-AG/1-AG	OEA	SEA	PEA
Maternal CM load	*r_S_*	0.005	*0.158*	−0.045	−0.313***	−0.113	0.081	0.008	0.008	−0.098	−0.089
	*p*	0.974	0.054	0.585	⩽ 0.001	0.170	0.432	0.921	0.921	0.240	0.284
		Children, *N* = 92					Children, *N* = 170				
		AEA	2-AG/1-AG	OEA^d^	SEA	PEA	AEA^c^	2-AG/1-AG	OEA	SEA	PEA
Maternal CM load	*r_S_*	–	0.147	0.134	0.028	0.012	−0.023	0.086	−0.046	−0.151*	−0.044
	*p*	–	0.163	0.203	0.789	0.911	0.830	0.266	0.549	0.049	0.568

*Note*: * *p* < 0.050, ** *p* < 0.010, *** *p* < 0.001, two-tailed. Underlined *p* values are significant after correction with false discovery rate (FDR). Italic *p* values indicate a trend for significance (*p* < .100). Bivariate correlations were computed with the maximal number of cases available: ^a^*n* = 39; ^b^*n* = 97; ^c^*n* = 88. Note that no AEA levels were detectable in the hair of children at *t*_0_. Exposure to childhood maltreatment (CM) was assessed with the Childhood Trauma Questionnaire (CTQ, Bader et al., 2009).

d Considering the CTQ subscales, OEA significantly correlated with maternal emotional neglect even after FDR correction (see online Supplementary Table S5).

#### Infant hair

There were no significant correlations between maternal CM load and the eCB and NAE levels in infant hair at *t*_0_ and *t*_2_ (Table 2), except for a negative association of maternal CM load with infant SEA concentrations at *t*_2_, which was not significant after FDR correction. However, exploring the relevance of CM subtypes revealed that children of mothers with higher emotional neglect showed significantly higher OEA concentrations at *t*_0_, which remained significant after FDR correction (see online Supplementary Table S5).

### Alterations in eCB and NAE levels within the first year postpartum

**Figure 1.**
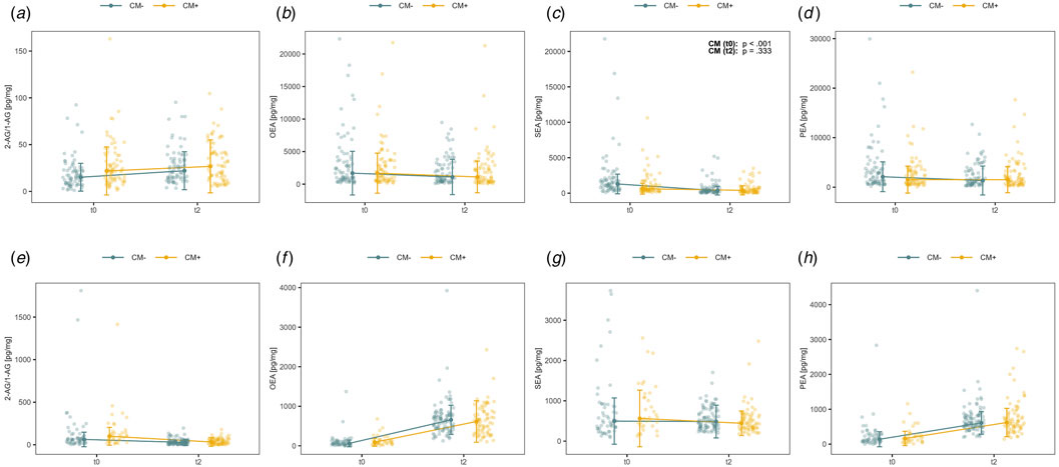
Course of endocannabinoids depending on maternal CM. Endocannabinoid (eCB) and *N*-acylethanolamines (NAE) hair concentrations (pg/mg) in mothers (a–d; *N_t0_* = 150, *N_t2_* = 148) and their children (e–h; *N_t0_* = 92, *N_t2_* = 170) with lower (CM−) and higher childhood maltreatment (CM+) load representing last trimester of pregnancy and 12 months postpartum. *t*_0_ hair sampled shortly after birth, representing the last trimester of pregnancy; *t*_2_ hair sampled 12 months postpartum, representing 10 to 12 months postpartum. Depicted in the upper right corner are *p*-values of significant *post hoc* tests of Time × CM load interactions. 2-AG/1-AG 2-arachidonoylglycerol, SEA stearoylethanolamide, OEA oleoylethanolamide, PEA palmitoylethanolamide.

We modeled alterations of eCB and NAE levels within the first year postpartum while considering potential effects of maternal CM load using robust linear mixed effects models. Tables 3 and 4 summarize the statistical results and Fig. 1 displays the findings for maternal (Fig. 1a–d) and infant hair (Fig. 1e–h). AEA levels could not be analyzed as too many measures were below the detection limit.

**Table 3.** Results of robust linear mixed effects models for endocannabinoid concentrations in mothers (*N_t0_* = 150; *N _t2_* = 148) ^a^

Outcome	Predictor	*b*	95% CI (*b*)	β	η^2^_p_ (95% CI)	*t*	*p*	*Post hoc* tests^1^		
								Compared groups	*b* (s.e.)	*p_FDR_*
2-AG/1-AG	Intercept	15.02	7.55–22.50	−0.27	0.05 (0.01–0.11)	3.94	< 0.001***			
	Time	10.52	−0.77–21.81	0.26	0.01 (0.00–0.05)	1.83	0.005**			
	CM	0.16	−0.04–0.36	0.10	0.00 (0.00–0.04)	1.58	0.113			
	Time × CM	−0.09	−0.45–0.16	−0.09	0.00 (0.00–0.03)	−0.92	0.357			
	Model statistics: conditional *R*^2^ = 0.036, marginal *R*^2^ = 0.036, *σ*_ri_ = 16.376; RMSE = 20.929									
OEA	Intercept	2436.32	1514.46–3358.19	−0.13	0.08 ( 0.03–0.15)	5.18	0.008**			
	Time	98.61	−1293.57–1490.78	−0.12	0.00 (0.00–0.01)	0.14	*0*.*080*			
	CM	−5.89	−30.45–18.68	−0.02	0.00 (0.00–0.02)	−0.47	0.639			
	Time × CM	−14.89	−52.59–22.80	−0.05	0.00 (0.00–0.02)	−0.77	0.439			
	Model statistics: conditional *R*^2^ = 0.018, marginal *R*^2^ = 0.018, *σ*_ri_ = 2019.904; RMSE = 3534.012									
SEA	Intercept	1605.40	1290.08–1920.72	−0.05	0.25 (0.17–0.33)	5.18	0.054	CM load at *t*_0_	−14.82 (4.29)	< 0.001***
	Time	−926.03	−1402.21 – −449.84	−0.27	0.05 (0.01–0.10)	0.14	< 0.001***	CM load at *t*_2_	−4.83 (4.99)	0.333
	CM	−14.82	−23.22 – −6.42	−0.09	0.04 (0.01–0.09)	−0.47	0.001**	^2^CM_low_ *t*_0_ v. *t*_2_	705 (116.7)	< 0.001***
	Time × CM	9.99	−2.90–22.88	0.06	0.00 (0.00–0.04)	−0.77	0.*054*	^2^CM_mean_ *t*_0_ v. t_2_	578 (82.1)	< 0.001***
	Model statistics: conditional *R*^2^ = 0.179, marginal *R*^2^ = 0.179, *σ*_ri_ = 690.901; RMSE = 2084.295							^2^CM_high_ *t*_0_ v. *t*_2_	452 (117.0)	< 0.001***
PEA	Intercept	2855.75	1897.47–3814.03	−0.15	0.10 ( 0.05–0.17)	9.98	0.002**			
	Time	65.19	−1381.97–1512.36	−0.08	0.00 (0.00–0.01)	−3.81	0.250			
	CM	−12.55	−38.08–12.99	−0.04	0.00 (0.00–0.03)	−3.46	0.335			
	Time × CM	−10.13	−49.31–29.06	−0.03	0.00 (0.00–0.02)	1.50	0.612			
	Model statistics: conditional *R*^2^ = 0.015, marginal *R*^2^ = 0.015, *σ*_ri_ = 2099.693; RMSE = 3749.330									

*Note.* * *p* < 0.050, ** *p* < 0.010, *** *p* < 0.001, two-tailed. Italic *p* values indicate a trend for significance (*p* < .100). All models include random intercepts to consider repeated measures within individuals (σri presents the standard deviation of random intercepts across all subjects). Coefficients of determination (*conditional R^2^*) present the variance explained by the total model (fixed and random effects) and *marginal R^2^* the variance explained by fixed effects only; RMSE presents the absolute model-to-data-fit by estimating the unexplained variance (quantified deviation of the estimated from the predicted values). Overall model tests cannot be calculated for robust linear mixed effects models. Exposure to childhood maltreatment (CM) was assessed with the sum score of the Childhood Trauma Questionnaire (CTQ, Bader et al., 2009).

a Note that data on eCB and NAE concentrations at both, *t*_0_ and *t*_2_, were available from 63 mothers only. Considering only these cases did not change the pattern of results (see online Supplementary Table S10 & Fig. S7).

1 *Post hoc* tests were performed to describe the nature of the significant interaction effects. *p* values were estimated from *z* statistics and adjusted for multiple comparisons with the false discovery rate (FDR).

^2^For *post hoc* contrasts between measurement points (*t*_0_ v. *t*_2_) CM was grouped in three CM severity groups based on the mean CTQ sum score (CM_mean_) as well as one s.d. below (CM_low_) or above (CM_high_) the average CTQ sum score.

**Table 4.** Results of robust linear mixed effect models for endocannabinoid concentrations in children (*N_t0_* = 92; *N _t2_* = 170)^a^

Outcome	Predictor	*b*	95% CI (*b*)	*β*	*η^2^*_p_ (95% CI)	*t*	*p*
2-AG/1-AG	Intercept	80.89	56.63–105.16	0.01	0.06 (0.01–0.12)	6.53	0.816
	Time	−47.02	−77.51 −16.52	−0.25	0.01 (0.00–0.05)	−3.02	<0.001***
	CM load	−0.05	−0.73 0.63	−0.00	0.00 (0.00–0.05)	−0.15	0.882
	Time × CM	0.11	−0.74 0.95	0.01	0.00 ( 0.00–0.03)	0.25	0.806
	Model statistics: conditional *R*^2^ = 0.194, marginal *R*^2^ = 0.194, *σ*_ri_ = 42.364; RMSE = 173.393						
OEA	Intercept	65.55	−67.84 198.94	−0.84	0.10 (0.00–0.17)	0.96	<0.001***
	Time	511.49	343.86–679.12	1.15	0.0 (0.19–0.01)	5.98	<0.001***
	CM load	1.11	−2.62 4.85	0.03	0.00 (0.00–0.02)	0.58	0.560
	Time × CM	0.16	−4.47 4.79	0.00	0.00 (0.00–0.03)	0.07	0.946
	Model statistics: conditional *R*^2^ = 0.533, marginal *R*^2^ = 0.533, *σ*_ri_ = 232.880; RMSE = 362.045						
SEA	Intercept	530.87	353.21–708.53	−0.07	0.28 (0.19–0.37)	5.86	0.282
	Time	34.06	−189.20 257.33	−0.15	0.05 (0.01–0.12)	0.30	*0.058*
	CM load	1.81	−3.16 6.79	0.05	0.04 (0.01–0.10)	0.71	0.475
	Time × CM	−3.29	−9.46 2.88	−0.08	0.00 (0.00–0.05)	−1.04	0.296
	Model statistics: conditional *R*^2^ = 0.018, marginal *R*^2^ = 0.018, *σ*_ri_ = 310.159; RMSE = 522.915						
PEA	Intercept	197.85	55.13–340.57	−0.71	0.12 (0.05–0.20)	2.72	<0.001***
	Time	417.36	238.00–596.71	0.88	0.00 (0.00–0.01)	4.56	<0.001***
	CM load	0.16	−3.83 4.16	0.00	0.00 (0.00–0.03)	0.08	0.937
	Time × CM	0.88	−4.08 5.84	0.02	0.00 (0.00–0.02	0.35	0.728
	Model statistics: conditional *R*^2^ = 0.426, marginal *R*^2^ = 0.426, *σ*_ri_ = 249.165; RSME = 454.858						

*Note.* * *p* < 0.050, ** *p* < 0.010, *** *p* < 0.001, two-tailed. Italic *p* values indicate a trend for significance (*p* < .100). All models include random intercepts to consider repeated measures within individuals (*σ*_ri_ standard deviation of random intercepts across all subjects). Coefficients of determination (*conditional R^2^*) present the variance explained by the total model (fixed and random effects) and *marginal R^2^* the variance explained by fixed effects only. RMSE presents the absolute model-to-data-fit by estimating the unexplained variance (quantified deviation of the estimated from the predicted values). Overall model tests cannot be calculated for robust linear mixed effects models. Exposure to childhood maltreatment (CM) was assessed with the sum score of the Childhood Trauma Questionnaire (CTQ; Bader et al., 2009).

a Note that data on eCB and NAE concentrations at both, *t*_0_ and *t*_2_, were available from 45 children only. Considering only these cases did not change the pattern of results (see online Supplementary Table S11 & Fig. S7).

#### Maternal hair

2-AG/1-AG levels significantly increased in maternal hair over the first year postpartum (*t*_Time_ = 1.83, *p* = 0.005, η^2^_p_ = 0.01; Table 3), which did not depend on maternal CM load (*t*_Interaction_ = −0.92, *p* = 0.357, η^2^_p_ < 0.01). There was no main effect of maternal CM load (*t*_CM_ = 1.58, *p* = 0.113, η^2^_p_ < 0.01). Conversely, SEA concentrations in maternal hair significantly decreased over time (*t*_Time_ = 0.14, *p* < 0.001, η^2^_p_ = 0.05), and were significantly lower in mothers with higher CM load (*t*_CM_ = −0.47, *p* = 0.001, η^2^_p_ < 0.04; see Fig. 1c). A marginally significant interaction of Time × Maternal CM load (*t*_Interaction_ = −0.77, *p* = 0.054, η^2^_p_ < 0.01) indicated that women with higher CM load had lower SEA level than women with lower CM load at *t*_0_ (*p_FDR_* < 0.001), while these differences were not found at *t*_2_ (*p_FDR_* = 0.333). No significant effects were observed for maternal OEA and PEA concentrations.

#### Infant hair

Infants exhibited an inversed pattern of change as compared to their mothers. From the last trimester of pregnancy to one year later, the 2-AG/1-AG concentration in the hair of infants decreased significantly (*t*_Time_ = −3.02, *p* < 0.001, η^2^ = 0.01), whereas OEA, PEA, and SEA levels increased significantly (see Table 4). There were no significant main or interaction effects of maternal CM load.

Limiting the analyses to mothers (*N* = 63) and children (*N* = 45) with complete data at both timepoints did not change the pattern of results (see online Supplementary Tables S10 and S11 and Fig. S7).

## Discussion

We investigated longitudinal alterations in eCB and NAE hair concentrations of women with varying degrees of CM and their children using hair samples representing the last trimester of pregnancy and one year after birth. The investigated biomarkers indicated changes in the activity of the eCB system from late pregnancy to one year later in both mothers and children. In late pregnancy, maternal CM accounted for differences in maternal eCB and NAE levels, while these alterations could not be found at one year postpartum. Thus, the effects of CM on the eCB system appear to be limited to the pre- and perinatal period and do not persist until one year later.

### eCB and NAE levels in maternal hair at the perinatal and postpartum period

Independent of maternal CM, 2-AG/1-AG concentrations in maternal hair were lower in late pregnancy than one year postpartum, while SEA concentrations decreased from late pregnancy to one year postpartum. Our findings indicate that the activity of the eCB system undergoes alterations during pregnancy and subsequent recovery, which extends initial findings of intra-individual variation during pregnancy (Krumbholz et al., 2013). The observed alterations suggest that the eCB system is differentially regulated during pregnancy and postpartum, which might influence the regulation of the glucocorticoid and immune system in these periods.

During pregnancy, HPA-axis functioning is altered resulting in progressively increasing tonic glucocorticoid secretion, which is suppressed around delivery (Brunton, Russell, & Douglas, 2008; Jung et al., 2011; Mastorakos & Ilias, 2003). 2-AG critically regulates HPA-axis activity as a turn-off signal in the HPA axis' negative feedback loop, ending further glucocorticoid secretion (Hill & Tasker, 2012). Our study indicates lowered 2-AG/1-AG levels during late pregnancy which may contribute to reduced negative feedback in the HPA axis, and thus to an increase in tonic cortisol levels in the last pregnancy trimester. Increasing 2-AG/1-AG hair levels from pregnancy to one year postpartum might reflect the restoration of a tighter 2-AG-mediated HPA-axis regulation that returns glucocorticoid secretion back to pre-pregnancy levels. Suiting our interpretation, Krumbholz et al. (2013) reported negative associations of glucocorticoid and 2-AG/1-AG concentrations in maternal hair over the course of pregnancy and parturition.

Furthermore, successful pregnancy depends on the tuned regulation of eCB and NAE in tissues of the reproductive system (Kozakiewicz et al., 2021; Maia et al., 2020; Schuel et al., 2002). Thereby, the immunomodulating properties of eCB and NAE are involved in the time- and tissue-specific (e.g. placenta, fetal membranes) regulation of inflammatory activity during pregnancy and birth (Mor, Cardenas, Abrahams, & Guller, 2011; Taylor et al., 2010). Disruptions in this regulation are associated with failure of implanting the inseminated ovum, impaired fetal development, premature birth, and even miscarriage (El-Talatini et al., 2009; Fonseca et al., 2010a, 2010b; Gebeh et al., 2013; Maia et al., 2020). Considering its immunomodulatory properties (Dalle Carbonare et al., 2008; Kasatkina, Heinemann, Hudz, Thomas, & Sturm, 2020; Tsuboi et al., 2018), increased SEA levels observed in late pregnancy could represent a regulatory signal of the body to govern the immunological processes in pregnancy (Corwin, Bozoky, Pugh, & Johnston, 2003; Maes, Ombelet, De Jongh, Kenis, & Bosmans, 2001; Taylor et al., 2010). To substantiate these preliminary interpretations, further research needs to elucidate the role of eCBs and NAEs in pregnancy and to investigate whether hair-based biomarkers of the eCB system could inform about clinically relevant pregnancy outcomes.

### Influence of maternal CM exposure on eCB and NAE levels over the first year postpartum

While all women showed decreasing SEA and increasing 2-AG/1-AG levels from late pregnancy to one year later, women with a history of CM exhibited significantly lower SEA levels in late pregnancy as compared with women without CM. This supports our previous results of lower SEA and higher 1-AG levels in hair of mothers with a history of CM (Koenig et al., 2018b) and reduced levels of SEA in highly traumatized civil war survivors with PTSD (Wilker et al., 2016). Given the role of SEA in modulating inflammatory and pain processes as well as in regulating glucocorticoid secretion, lowered SEA levels in the hair of pregnant women with a CM history may implicate that CM contributes to an aberrant immune and HPA axis (re-)activity in late pregnancy through altered SEA regulation. Indeed, there is evidence that women with CM history exhibit higher inflammatory activity during pregnancy (Boeck et al., 2016; Bublitz, De La Monte, Martin, Larson, & Bourjeily, 2017, 2022; Kleih et al., 2022). NAEs are known to inhibit inflammation by binding to peroxisome proliferator-activated-receptors (PPAR) (O'Sullivan & Kendall, 2010), and therefore a reduced SEA signaling may contribute to increased inflammation in pregnant women with a history of CM.

Most importantly, our study is the first to show that the associations between maternal CM and SEA levels no longer exist one year postpartum and that maternal CM history did not account for differences in the eCB markers investigated. About one year after birth, the regulation of the immune and glucocorticoid system has conceivably returned to a pre-pregnancy state, and correspondingly, pregnancy-related regulatory alterations in the eCB system will have ‘normalized’. In such a condition, i.e., in the absence of a particular physiological stressor (e.g. due to pregnancy), CM-related differences in the regulation of the eCB system do not appear to be present. Thus, CM-affected women may not differ from non-CM-affected women in their basal eCB system activity, but the eCB system rather differs in its reactivity to the physiological challenge of pregnancy. This pattern of results fits with the perspective that consequences of early adversity on the regulation of physiological systems do not necessarily show as permanent changes in tonic activity, but specifically manifest as a higher reactivity upon exposure to psychosocial, immunological, and physiological stressors (Danese & Baldwin, 2017). Underlining this, previous studies indicated that the stress-induced increase in inflammatory activity is higher in CM-exposed individuals than in nonexposed individuals (Danese & Baldwin, 2017; Fagundes, Glaser, & Kiecolt-Glaser, 2013), and it has also been shown that CM-exposed women presented an upregulated immune-cellular energy metabolism compared to non-exposed women directly after birth, while this effect was not detectable one year postpartum (Gumpp et al., 2022).

Altogether, we interpret our findings as evidence that CM exposure to contributes to a sensitization of biological stress response systems to psychosocial, immunological, and physiological stressors. This also means that the organism of pregnant women with a history of CM faces a higher compensatory/regulatory strain (i.e. allostatic load) at the same exposure to stress (e.g., pregnancy; Danese & McEwen, 2012; Fava et al., 2019). This might contribute to a higher risk for negative health outcomes after CM, including increased inflammatory reactions, more pain, and possibly more complications during pregnancy. Moreover, it could be that unborn children of CM-affected women are confronted with an altered physiological milieu *in utero*.

### eCB and NAE levels in infant hair at the perinatal and postpartum period

To investigate possible intergenerational effects of CM on the eCB system, we collected hair of infants to analyze the longitudinal course of eCB system markers from late pregnancy to one year postpartum. We provide first evidence for a general developmental change in eCB and NAE levels in infant hair: that is, 2-AG/1-AG levels decreased, while OEA, SEA, and PEA levels increased from the last trimester of pregnancy until one year later. Our findings of elevated 2-AG/1-AG levels in newborns resemble first evidence from animal studies showing that 2-AG concentrations peak in various tissues and biomaterials of neonate rodents during the perinatal period and subsequently decreased in the postpartum (Berrendero, Sepe, Ramos, Di Marzo, & Fernández-Ruiz, 1999; Ellgren et al., 2008; Fride, 2008; Lee & Gorzalka, 2012). Increased 2-AG presumably serves to induce suckling behavior directly after birth and to enhance (neuro)development and growth (Berrendero et al., 1999; Fride, 2004, 2008; Schuel et al., 2002). With our study we provide first evidence that 2-AG/1-AG might follow a similar trajectory in humans.

Moreover, we provide first data on the course of NAE levels in human newborns. Starting from substantially lower levels than in the mothers, the NAE concentrations in the hair of infants increased from late pregnancy to one year later approaching maternal levels. This resembles the trajectory of the biologically similar AEA in animals, which gradually increases throughout infancy (Lee & Gorzalka, 2012). Altogether, our data indicate that eCB and NAE levels fluctuate over different developmental stages in early life, which probably continues throughout maturation (Lee & Gorzalka, 2012; Meyer, Lee, & Gee, 2018). As pioneered with our study, future research may use hair samples of newborns to retrospectively assess eCB and NAE levels in unborn children and gain insight into prenatal development of the eCB system.

### Intergenerational effects of maternal CM on eCB and NAE levels in children

The change of eCB and NAE levels in children in the first year postpartum was not affected by maternal CM load. Exploring the relevance of CM subtypes revealed that maternal emotional neglect was linked to higher OEA levels in children in late pregnancy (see online Supplementary Table S5). As maternal and fetal eCB systems interact via the fetoplacental unit (Keimpema, Calvigioni, & Harkany, 2013), it might be that CM-associated alterations in children's OEA levels in late pregnancy result from an altered intrauterine milieu in mothers with a history of emotional neglect. It was previously shown that the emotional stress CM-affected women experience during pregnancy is linked to altered hormone concentrations in neonatal hair (Entringer, Buss, & Wadhwa, 2015; Hoffman, D'Anna-Hernandez, Benitez, Ross, & Laudenslager, 2017). Most importantly, we found no association between maternal CM and eCB/NAE levels in infant hair one year postpartum, indicating that possible intergenerational effects of CM on the children's eCB system resolved within the first year after birth. It remains to be investigated how possible intergenerational imprinting of the eCB system by maternal CM experiences affects the fetal development *in utero* and the physical, immunological, and mental development of children beyond their first year of life.

### Limitations

We investigated a rather homogenous group of predominantly healthy postpartum women, reporting mild to moderate CM experiences, mostly living in committed relationships, and with rather high education and good socioeconomic status. The nature of our study cohort might limit the generalizability of our findings and could underestimate the true impact of CM on the eCB system. Future studies should aim at sampling women with higher CM exposure. The data from both points of measurement could only be combined for a subsample, which limits the statistical power to detect small intergenerational effects. Moreover, it has been reported that eCB/NAE concentrations differ between biological specimens (e.g. tissue *v.* circulation; brain *v.* periphery; liquid *v.* keratin matrices) and systems (e.g. reproductive system *v.* brain) as well as along developmental trajectories (Fonseca et al., 2010a, 2010b). Although lipophilic basic compounds such as eCB are assumed to be incorporated from the bloodstream and bound to hair pigments such as melanin (Thieme, Anielski, Helfers, & Krumbholz, 2020), the incorporation of eCBs and NAEs into hair has not been finally understood (Krumbholz et al., 2013; Liu & Doan, 2019) and might not necessarily reflect tissue-dependent alterations (Fonseca et al., 2010b).

## Conclusion

Mothers and children showed alterations in the eCB system from the last trimester of pregnancy to one year postpartum. Maternal CM accounted for alterations in the eCB system of mothers, which were limited to late pregnancy but normalized in the first year after birth. Future research needs to investigate the regulation of the eCB system *in utero* and its relevance in mediating pregnancy outcomes, as well as intergenerational effects on the mental and physical development of children before and after birth.

## Supporting information

Hitzler et al. supplementary material Hitzler et al. supplementary material

## Footnotes

† The notes appear after the main text.

## Notes

1 In a previous publication (Koenig et al., 2018b), we used group comparison tests to analyze CM group-related differences in eCB and NAE levels in maternal and infant hair collected at *t*_0_. To this end, we had categorized mothers in none-mild CM *v.* moderate-severe CM exposure based on the mild clinical cut-off of the CTQ. For comparability of findings, we applied the mild CTQ cut-off to the data of the present study and provided group comparison tests in the online Supplementary (see online Supplementary Table S8 & S9 and Supplementary Figs S3–S6). In summary, all analytic approaches conclude that at *t*_0_, higher maternal CM exposure is associated with lower SEA and higher 2-AG/1-AG levels in maternal hair, and that in trend, levels of OEA and 2-AG/1-AG levels are higher in the hair of children of mothers with a history of CM exposure. All these associations vanished at one year after birth (see online Supplementary Table S8 & S9).

## Supplementary material

The supplementary material for this article can be found at https://doi.org/10.1017/S0033291723001204
