# Supplementary material for: Longitudinal course of endocannabinoids and N-acylethanolamines in hair of mothers and their children in the first year postpartum: investigating the relevance of maternal childhood maltreatment experiences
Source: Psychol Med. 2023 May 18;53(15):7446–57. doi: 10.1017/S0033291723001204 (PMC10719681; doi:10.1017/S0033291723001204)
Supplement: Hitzler et al. supplementary material [file S0033291723001204sup001.pdf]

-- Supplementary Material --

Longitudinal course of endocannabinoids and *N*-acylethanolamines in hair of mothers and their children in the first year postpartum: Investigating the relevance of maternal childhood maltreatment experiences

Melissa Hitzler[1*], Lynn Matits[1,2], Anja M. Gumpp[1], Alexandra M. Bach[1], Ute Ziegenhain[3], Wei Gao[4], Iris-Tatjana Kolassa[1,] Alexander Behnke[1]

[1]Clinical and Biological Psychology, Institute of Psychology and Education, Ulm University, Ulm, Germany

[2]Division of Sports and Rehabilitation Medicine, Department of Medicine, Ulm University Hospital, Ulm, Germany

[3]Department of Child and Adolescent Psychiatry, Ulm University Hospital, Ulm, Germany

[4]Department of Biopsychology, Technische Universität Dresden, Dresden, Germany

* Corresponding author:
M.Sc.-Psych. Melissa Hitzler
Melissa.hitzler@uni-ulm.de

<u>Content:</u>

Study Flow
Study Flow Hair Sampling
Description Hair Processing and Endocannabinoid Analysis
Supplementary Descriptive Data
Supplementary Statistical Analyses

Study Flow

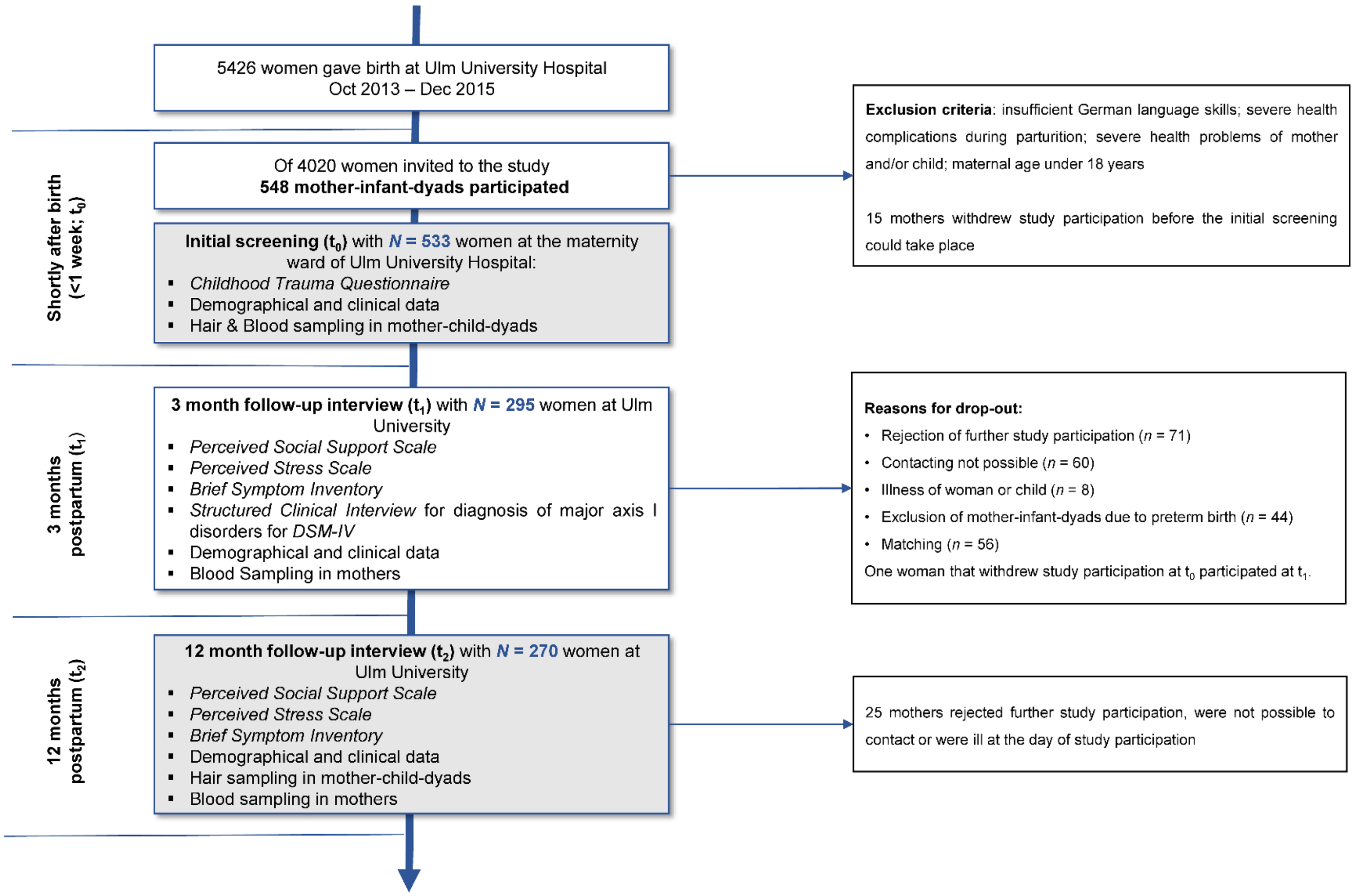

Supplementary Figure S1. Study Flow. Flow chart representing the procedures of recruitment, withdrawal rates, characterization and analyses of the study cohort in the project "My Childhood – Your Childhood". The exclusion of preterm births was chosen as further exclusion criteria at t_1 as child development should be analyzed. At t_1 mothers with and without CM were matched. Thus, N = 56 women without CM experiences were not contacted at t_1 due to this matching process.

Grey depicted points of measurement are included in the current study and the novel longitudinal analyses of t_0 and t_2 data.

Study Flow Hair Sampling Procedure

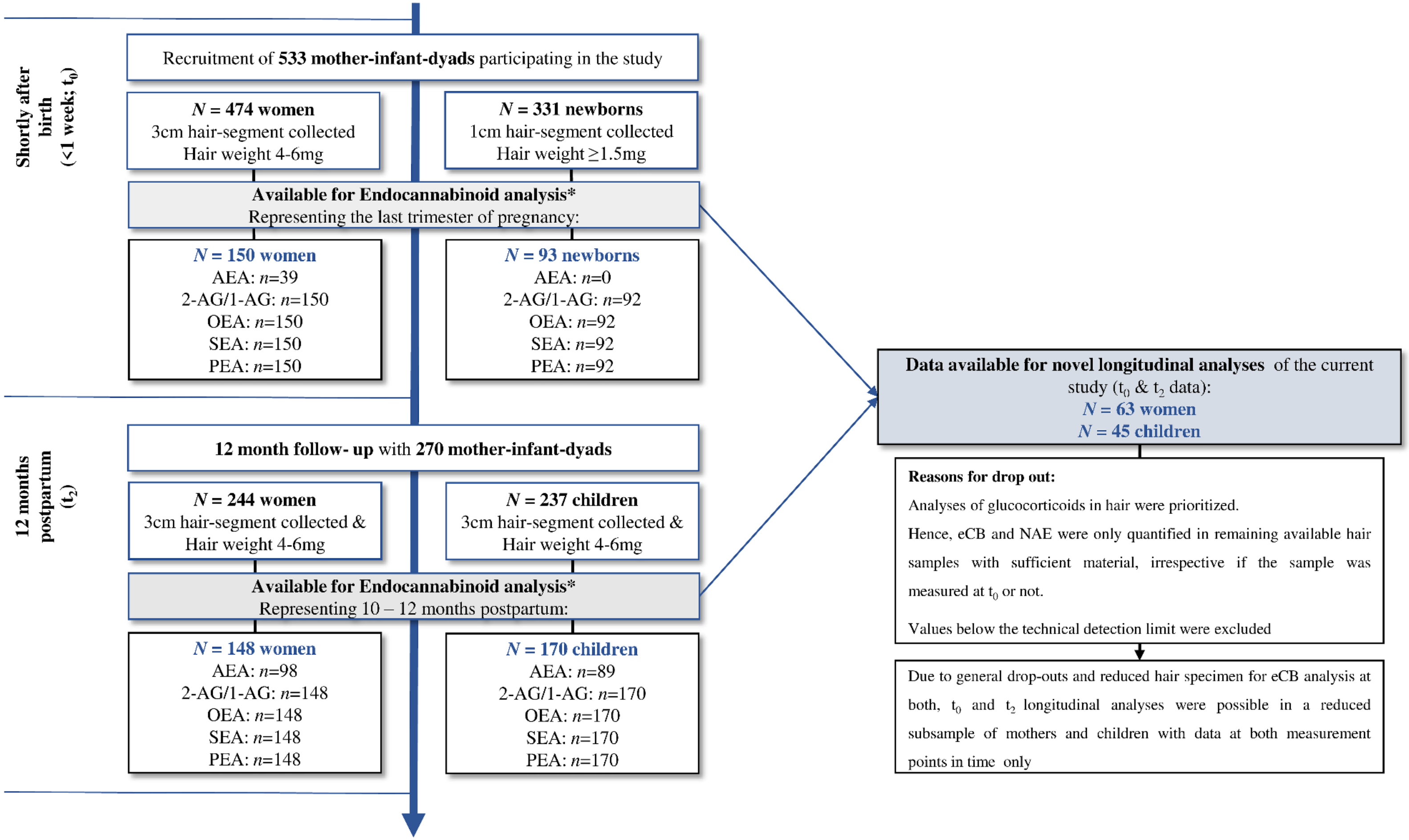

Supplementary Figure 2. Study flow and drop-out rates of hair sampling. Overview of recruitment and hair specimen collection after applying exclusion criteria and removal of missing values and values under technical detection limit.

Note. At t_0 *n* = 59 mother-child-dyads rejected hair sampling; *n* = 143 newborns had to spares hair or were too fidgety for sampling. At t_2 *n* = 26 mothers-child-dyads rejected hair sampling; *n* = 7 children had to spares hair or were too fidgety for hair sampling. For general drop-out rates and reasons between t_0, t_1 and t_2 see Supplementary Figure S1. Note that due to its low abundance in hair, the measurement of AEA led to many values below the detection limit and thus missing values for analysis. Except for AEA, full eCB/NAE data at both measurement points in time was available from *n* = 63 mothers and *n* = 45 children enabling longitudinal analyses.

* t_0 eCB data was previously published in Koenig et al., 2018. For reasons of comparability, the same cohort was considered in the current study using a different statistical approach; Results from Koenig et al., 2018 were replicated within the current study. t_2 eCB data and longitudinal analyses were considered for the first time in the current study. AEA anandamide; 2-AG/1-AG 2-arachidonoylglycerol; SEA stearoylethanolamide; OEA oleoylethanolamide; PEA palmitoylethanolamide.

Supplementary Information: Hair Processing and Endocannabinoid Analysis

While circulating eCB concentrations mirror a state and can fluctuate depending on circadian rhythmicity and acute stress (Vaughn et al., 2010), hair analyses provide the more reliable and stable measurement of eCB (Krumbholz et al., 2013), especially in the time around childbirth. Thus they allow for a retrospective measure of long-term eCB accumulation over weeks and months. Hair sampling took place directly after the psychodiagnostic interviews at t_0 and t_2 in mother-child-dyads that gave informed consent in sampling of biological specimen.

In total, hair was collected from $N = 474$ mothers and $N = 331$ children at t_0 and $N = 244$ mothers and $N = 237$ children at t_2. At t_0 59 mother-child-dyads rejected hair sampling. In $n = 143$ mothers declined hair sampling in their infants, the newborns hair was to spares for sampling or infants were too fidgety. At t_2 26 mother-child-dyads rejected hair sampling; $n = 7$ children had to spares hair or were too fidgety for hair sampling (for general drop out reasons between t_0, t_1 and t_2 see Figure S1). Upon limited material, the analysis of the primary hair-based biological marker, steroid hormones, was prioritized (data not shown here). At both times of measurement eCB and NAE were quantified in all remaining hair specimen with sufficient material. At t_0, sufficient material for additional eCB and NAE quantification was available for 150 mothers and 92 children; and at t_2, eCB and NAE were measured in the hair of 148 mothers and 170 children. However, due to study drop outs, reduced available hair samples for eCB analysis and eCB values under technical detection limit only a subsample of $N = 63$ mothers and $N = 45$ children presented complete eCB and NAE data at both measurement points in time (see Figure S2 for details).

Hair collection. At t_0 and t_2, hair samples were collected and processed by trained academic staff, using laboratory gloves to avoid contamination of the hair with skin moisture. In mothers, optimally three hair strands (~3mm diameter each) were cut close to the scalp from the posterior vertex position. When this sampling location was not possible for the children due to sparse hair, infant hair samples were cut from locations with most hair, usually at the hairline beneath the ear. After parturition (t_0), newborns' hair was washed with clear water to preclude contamination with blood or amniotic fluid. Hair samples were wrapped in aluminum foil and stored at −20 °C to minimize possible degradation effects and loss of biochemical compounds due to long-term storage and humidity. To avoid contamination of the hair with skin moisture, hair samples were collected and processed using laboratory gloves.

Pre-processing. Before shipment to the laboratory of Prof. Kirschbaum at TU Dresden for LC-MS/MS mass spectrometry analyses, the preprocessing of all hair samples was performed in the laboratory of Prof. Kolassa at Ulm University. First, hair strands of the same subject were pooled. In a standardized procedure, the 3cm-hair segment proximal to the scalp was cut. Due to an approximate adult hair growth of ~1 cm/month (Wennig, 2000), the 3cm-hair segment proximal to the scalp reflects maternal cumulative eCB concentration incorporated in the last three months. However, fetal/neonatal hair grows slower than in adults with ~1cm in during the three months of the third trimester of pregnancy (cf. Gareri & Koren, 2010). Thus, to display the metabolic activity during the last three months of prenatal development, hair of newborns collected at t_0 was cut to 1 cm segments. At t_2 both, the proximal 3cm-hair segment of mothers and children was used for analyses, reflecting month 10 to 12 postpartum. To our knowledge, studies of children's hair growth velocity, in particular for children aged around 12 months, are spares. First evidence suggests, that hair growth velocity increases over the first year after birth and aligns to the growth rate of adults (Pecoraro et al., 1964; de Kruiff et al., 2020): In a small sample of children aged 6 to 12 months hair growth velocity was around ~6.7 mm/ per month and with 12 to 24 months ~9.4 mm/ per month (c.f., de Kruiff et al., 2020). Thus, for eCB analysis one year after birth, for mothers and children likewise, the 3cm-hair segment proximal

to the scalp was used to reflect eCB levels of the same time period allowing to take a look into the first year postpartum. Cut hair of mothers and children was weighed (range 4–6mg) and placed into Falcon tubes. The non-pulverized hair of mothers had an average weight of 5.2 mg (*SD*=0.9) at t_0 and 5.2 mg (*SD* = 0.7) at t_2. Infant hair had an average weight of 2.4 mg (*SD*=1.7) at t_0 and 5.3 (*SD* = 0.5) at t_2. For further sample details and missing data see Figure 1 in the main manuscript.

Supplementary Descriptive Data

Supplementary Table S1. Endocannabinoid hair concentrations in pg/mg for all available data of mothers and children

		**Mothers t_0**			**Mothers t_2**		
		Whole Sample<br>*N* = 150	CM+<br>*n* = 76	CM-<br>*n* = 74	Whole Sample<br>*N* = 148	CM+<br>*n* = 74	CM-<br>*n* = 74
**AEA**	*Med (IQR)*	--	0.52 (0.38)[a]	--	0.41 (0.29)[b]	0.46 (0.28)[c]	0.39 (0.24)[d]
	M (SD) †	--	0.53 (0.32)	--	0.49 (0.36)	0.47 (0.20)	0.53 (0.47)
	Min	--	0.20	--	0.12	0.12	0.17
	Max	--	1.97	--	2.62	0.87	2.62
**2-AG/1-AG**	*Med (IQR)*	17.73 (18.19)	21.86 (25.82)	15.14 (15.50)	24.17 (25.66)	26.75 (29.06)	22.10 (50.59)
	M (SD) †	23.73 (21.43)	27.99 (24.42)	19.35 (16.93)	29.02 (20.29)	30.68 (21.62)	27.36(18.87)
	Min	1.13	1.50	1.13	3.47	5.73	3.47
	Max	163.01	163.01	92.54	104.63	104.63	95.25
**OEA**	*Med (IQR)*	1687.52 (3124.05)	1685.82 (3110.54)	1687.52 (3474.40)	1097.90 (2573.43)	1108.46 (2520.21)	1096.89 (2783.47)
	M (SD) †	3163.32 (4078.56)	2885.19 (3669.16)	3448.96 (4467.44)	2197.41 (2747.61)	2271.74 (3219.87)	2123.09 (2196.23)
	Min	139.44	144.51	139.44	78.52	78.52	112.30
	Max	22323.53	21756.43	22323.53	21283.02	21283.02	9479.25
**SEA**	*Med (IQR)*	974.01 (1318.88)	623.94 (791.21)	1297.11 (1431.19)	375.61 (643.51)	397.64 (650.52)	344.24 (625.55)
	M (SD†*)*	1731.60 (2761.85)	1179.34 (1611.24)	2298.78 (3501.17)	663.81 (885.46)	655.77 (793.98)	671.84 (973.80)
	Min	91.54	91.54	105.43	19.38	37.93	19.38
	Max	21768.91	10620.49	21768.91	5194.78	3507.43	5194.78
**PEA**	*Med (IQR)*	1934.14 (3006.46)	1529.50 (2734.35)	2103.40 (3189.15)	1473.98 (2942.41)	1509.36 (2784.92)	1343.32 (3040.59)
	M (SD) †	3352.48 (4379.27)	2836.66 (3515.97)	3882.234(5087.43)	2527.90 (2869.33)	2676.39 (3207.29)	2469.41 (2504.09)
	Min	59.66	59.66	192.86	263.70	263.70	264.62
	Max	29949.58	23215.63	29949.58	17660.38	17660.38	12679.25

		Children t_0			Children t_2		
		Whole Sample *N* = 92	CM+ *n* = 37	CM- *n* = 55	Whole Sample *N* = 170	CM+ *n* = 76	CM- *n* = 94
**AEA**	*Med (IQR)*	--	--	--	0.28 (0.15)[e]	0.25 (0.17)[f]	0.28 (0.14)[g]
	M (SD) †	--	--	--	0.30 (0.13)	0.30 (0.14)	0.29 (0.12)
	Min	--	--	--	0.08	0.08	0.11
	Max	--	--	--	0.75	0.75	0.63
**2-AG/1-AG**	*Med (IQR)*	72.62 (101.67)	100.05 (114.00)	62.72 (95.63)	30.66 (44.81)	33.43 (50.22)	27.54 (35.90)
	M (SD) †	151.6 (280.55)	162.15 (237.97)	143.79 (307.81)	38.42 (34.74)	41.29(34.89)	35.89 (34.72)
	Min	4.27	11.20	4.27	0.85	1.80	0.85
	Max	1810.29	1414.29	1810.29	196.08	179.26	196.08
**OEA**	*Med (IQR)*	74.64 (89.74)	91.32 (100.63)	61.66 (90.89)	641.72 (410.31)	614.81 (534.86)	657.12 (376.28)
	M (SD) †	118.01 (173.88)	133.12 (136.64)	107.84 (195.55)	674.37 (427.96)	652.33 (386.03)	691.89 (462.11)
	Min	0.82	12.08	.82	104.44	104.44	127.02
	Max	1371.86	682.63	1371.86	3923.08	2429.63	3923.08
**SEA**	*Med (IQR)*	522.62 (582.29)	562.89 (754.00)	497.53 (595.41)	461.25 (346.15)	443.70 (325.19)	485.90 (416.06)
	M (SD) †	786.24 (748.00)	790.89 (606.89)	783.11 (835.05)	542.18 (314.76)	521.00 (352.21)	559.94 (283.86)
	Min	94.35	128.33	94.35	157.33	157.33	202.96
	Max	3733.71	2559.86	3733.71	2479.16	2479.16	1703.70
**PEA**	*Med (IQR)*	151.36 (219.20)	162.38 (218.21)	139.76 (220.18)	610.88 (349.51)	620.11 (429.01)	608.65 (30.41)
	M (SD) †	247.33 (344.57)	245.26 (251.70)	248.73 (397.29)	738.28 (497.71)	759.15 (513.30)	719.69 (489.33)
	Min	0.25	2.23	0.25	142.28	142.28	200.53
	Max	2833.29	1159.71	2833.29	4400.00	2740.74	4400.00

Note: At t_0, AEA was analyzed in a subsample of mothers only, due to its low abundance in hair AEA measures of many cases were below the technical detection limit. The maximal number of available cases varies: [a]*n* = 39; [b]*n* = 98; [c]*n* = 53; [d]*n* = 44; [e]*n* = 89; [f]*n* = 43; [g]*n* = 45.

†As endocannabinoid data is skewed and not normal distributed, *M* and *SD* are no reliable measures and are only reported for reasons of comparability. For analysis and interpretation *Med* and *IQR* are used exclusively. According to the mild cut-off criterion of the CTQ (Bernstein & Fink, 1998), women without any CM experiences were classified as CM− and those with at least mild CM experiences in at least one CTQ subscale were categorized as CM+. CM Childhood Maltreatment; CTQ Childhood Trauma Questionnaire AEA anandamide, 2-AG/1-AG 2/1-arachidonoylglycerol; OEA N-oleoylethanolamide; SEA N-stearoylethanolamide; PEA N-palmitoylethanolamide.

Supplementary Table S2. Raw data of endocannabinoid hair concentration in pg/mg for *N* = 63 mothers and *N* = 45 children with hair data at both points of measurement

		Mothers t_0			Mothers t_2		
		Whole Sample (*N* = 63)	CM+ *N* = 37	CM- *N* = 26	Whole Sample (*N* = 63)	CM+ *N* = 37	CM- *N* = 26
**2-AG/1-AG**	*Med (IQR)*	20.73 (18.43)	21.77 (24.50)	14.04 (19.36)	27.78 (25.85)	30.81 (37.37)	24.85 (21.09)
	M (SD) †	25.09 (26.01)	28.16 (28.79)	20.73(21.24)	33.22 (22.11)	36.09 (24.12)	29.13 (18.56)
	Min	1.50	1.50	1.61	3.47	5.73	3.47
	Max	163.01	163.01	92.54	104.63	104.63	79.91
**OEA**	*Med (IQR)*	1805.63 (3115.71)	1805.63 (2616.89)	1883.03 (3265.46)	1015.93 (3034.79)	1002.38 (2897.88)	1075.10 (3277.85)
	M (SD) †	2944.00 (3826.27)	2423.30 (2499.37)	3684.99 (5132.27)	2395.73 (3435.95)	2436.83 (4010.14)	2337.23 (2472.90)
	Min	139.44	144.51	139.44	130.460	130.46	166.41
	Max	22323.53	10711.90	22323.53	21283.02	21283.02	8459.03
**SEA**	*Med (IQR)*	907.88 (1309.06)	677.23 (559.81)	1297.11 (1602.96)	479.25 (944.74)	479.25 (753.50)	514.65 (899.65)
	M (SD†)	1672.31 (2917.95)	1070.97 (1197.24)	2528.06 (4213.66)	943.64 (1120.17)	833.44 (955.28)	1100.47 (1324.47)
	Min	91.54	91.54	253.69	43.61	43.61	216.58
	Max	21768.91	6102.38	21768.91	5194.78	3507.43	5194.78
**PEA**	*Med (IQR)*	2027.14 (2718.37)	1495.13 (2575.54)	2504.12 (4424.66)	1343.57 (3479.15)	1535.38 (3526.70)	1334.58 (4127.04)
	M (SD) †	3178.00 (4378.29)	2408.12 (2568.27)	4273.61 (5993.03)	2731.58 (3188.12)	2740.06 (3548.94)	2719.51 (2658.95)
	Min	59.66	59.66	531.08	311.14	325.79	311.140
	Max	29949.58	12247.71	29949.58	17660.38	17660.38	10688.74

		Children t_0			Children t_2		
		Whole Sample (*N* = 45)	CM+ *N* = 21	CM- *N* = 24	Whole Sample (*N* = 45)	CM+ *N* = 21	CM- *N* = 24
**2-AG/1-AG**	*Med (IQR)*	62.72 (129.95)	74.30 (154.70)	60.89 (120.24)	11.31 (15.48)	10.69 (14.09)	12.29 (19.73)
	M (SD) †	135.35 (219.30)	178.35 (299.95)	97.72 (102.99)	15.05 (12.28)	12.95 (9.05)	16.88 (14.48)
	Min	6.84	18.70	6.84	0.85	2.14	0.85
	Max	1414.29	1414.29	377.51	59.51	32.48	59.51

**OEA**	*Med (IQR)*	70.71 (83.53)	91.32 (97.36)	57.59 (67.60)	429.52 (352.68)	407.65 (416.31)	504.08 (367.16)
	M (SD) †	106.69(117.90)	140.67 (1571.66)	76.96(54.79)	511.99 (265.75)	487.86 (291.91)	540.97 (243.18)
	Min	8.34	22.28	8.34	104.44	104.44	186.49
	Max	682.63	682.63	204.53	1176.89	1123.27	1176.89
**SEA**	*Med (IQR)*	461.21 (408.83)	474.20 (610.03)	378.31(426.23)	595.07 (358.82)	553. 73 (288.82)	597.46 (420.17)
	M (SD) †	577.24 (439.43)	671.68 (547.39)	494.61 (305.90)	639.00 (349.82)	626.39 (450.62)	650.04 (239.29)
	Min	122.82	128.33	122.82	226.29	280.19	226.29
	Max	2219.34	2219.34	1319.33	2479.16	2479.16	1065.95
**PEA**	*Med (IQR)*	143.79 (191.76)	153.21 (205.88)	132.18 (180.13)	503.16 (259.14)	508.18 (304.40)	499.70 (234.09)
	M (SD) †	207.03 (198.70)	243.07 (261.30)	175.49 (118.05)	558.85 (371.40)	589.91 (500.87)	531.67 (209.95)
	Min	20.07	32.29	20.07	216.70	225.74	216.70
	Max	1159.71	1159.71	527.33	2653.87	2653.87	1139.75

Note: At t_0, AEA was analyzed in a subsample of mothers only, due to its low abundance in hair AEA measures of many cases were below the technical detection limit.

†As endocannabinoid data is skewed and not normal distributed, *M* and *SD* are no reliable measures and are only reported for reasons of comparability. For analysis and interpretation *Med* and *IQR* are used exclusively. According to the mild cut-off criterion of the CTQ (Bernstein & Fink, 1998), women without any CM experiences were classified as CM− and those with at least mild CM experiences in at least one CTQ subscale were categorized as CM+.

CM Childhood Maltreatment; CTQ Childhood Trauma Questionnaire AEA anandamide, 2-AG/1-AG 2/1-arachidonoylglycerol; OEA N-oleoylethanolamide; SEA N-stearoylethanolamide; PEA N-palmitoylethanolamide.

Supplementary Table S3. Intercorrelations of endocannabinoids at each point of measurement.

Bivariate Spearman rank correlations (r_S) between endocannabinoid concentrations in mothers and children

	Mothers t_0 (N = 150)				Children t_0 (N = 92)					Intergenerational correlation t_0[f#]				
	AEA[a]	2-AG/1-AG	OEA	SEA	AEA	2-AG/1-AG	OEA	SEA		AEA	2-AG/1-AG	OEA	SEA	PEA
2-AG/1-AG	.12				--				2-AG/1-AG	--	.02	.07	.01	.09
OEA	.12	.21*	--	--	--	.72***			OEA	--	-.08	.17	-.08	.12
SEA	.21	-.03	.78***	--	--	.38***	.56***		SEA	--	-.14	.12	-.15	.06
PEA	.16	.17	.94***	.86***	--	.48***	.77***	.74***	PEA	--	.13	.17	-.02	.17
	Mothers t_2 (N =148)				Children t_2 (N = 170)					Intergenerational correlation t_2[g#]				
	AEA[b]	2-AG/1-AG	OEA	SEA	AEA[c]	2-AG/1-AG	OEA	SEA		AEA	2-AG/1-AG	OEA	SEA	PEA
AEA	--				--	--			AEA	.10[e]	.15	.11	.07	-.01
2-AG/1-AG	.28*				.09	--			2-AG/1-AG	.15[d]	-.13	.05	.09	-.03
OEA	.11	.16			.27*	.46**			OEA	-.02[d]	-.05	.06	.05	.07
SEA	.02	.14	.66***		-.01	-.22*	.31***		SEA	-.07[d]	-.44**	-.18	.20	-.18
PEA	.03	.08	.93***	.77***	-.01	.45***	.74***	.37***	PEA	-.02[d]	-.09	-.01	.06	.08

Note: * $p < .050$, ** $p < .010$, *** $p < .001$, two-tailed. All p-values were adjusted for multiple comparisons using the false discovery rate (FDR). Note that at t_0, AEA was analyzed in a subsample of mothers only. Due to its low abundance in hair the AEA measures of many cases were below the technical detection limit. The maximal number of available cases varies: [a]n = 39; [b]n = 98; [c]n = 89; [d]n = 62 mother-child-dyads; [e]n = 58 mother-child-dyads; [f]n = 62 mother-child-dyads; [g]n = 107 mother-child-dyads. [#] Maternal values depicted in rows and children's values depicted in columns.

AEA anandamide; 2-AG/1-AG 2/1-arachidonoylglycerol; OEA *N*-oleoylethanolamide; SEA *N*-stearoylethanolamide; PEA *N*-palmitoylethanolamide.

Supplementary Table S4 Bivariate Spearman rank correlations *(r*S) between endocannabinoid concentrations in hair sampled at t_0 and t_2 in *N* = 63 mothers and *N* = 45 children

		Mothers					Children				
		**t_2**					**t_2**				
		**AEA**	**2-AG/1-AG**	**OEA**	**SEA**	**PEA**	**AEA**	**2-AG/1-AG**	**OEA**	**SEA**	**PEA**
**t_0**	**AEA**	.29[a]	.05[a]	.36[a]	-.11[a]	.20[a]	--	**--**	--	--	--
	**2-AG/1-AG**	-.37[b]	.09	.03	-.30	.04	--	-.12	-.08	-.06	-.16
	**OEA**	.25[b]	-.02	**.41****	.25	**.34***	**--**	.00	.06	-.04	.02
	**SEA**	.41[b]	.03	**39****	**.43***	**.35***	--	.09	-.02	-.01	-.03
	**PEA**	.29[b]	.02	**.41****	.25	**.35***	--	.08	-.01	.06	.07

Note: * $p < .050$, ** $p < .010$, *** $p < .001$, two-tailed. All p-values were adjusted for multiple comparisons with the false discovery rate (FDR).
data: [a] *N* = 17 mothers; [b] *N* = 23; no AEA data available for children.

Supplementary Table S5 Bivariate Spearman rank correlations of maternal childhood maltreatment exposure with endocannabinoids measured in maternal hair and infant hair

	Hair samples collected shortly after parturition (t_0) representing the last trimester of pregnancy						Hair samples collected 12 months postpartum (t_2) representing 10-12 months postpartum					
	Mothers *N* = 150						Mothers *N* = 148					
	CM load	Emotional abuse	Physical abuse	Sexual abuse	Emotional neglect	Physical neglect	CM load	Emotional abuse	Physical abuse	Sexual abuse	Emotional neglect	Physical neglect
AEA	.005	-.007	-.170	-.153	*.269*	-.132	.081	.140	.046	.011	.062	-.006
2-AG/1-AG	*.158*	.115	.067	.054	.134	.037	.008	.119	.134	-.084	-.006	-.089
OEA	-.045	-.055	-.015	-.118	-.068	-.048	.008	.119	.134	-.084	-.006	-.089
SEA	**-.313*****	**-.224****	*-.143*	*-.156*	**-.271****	**-.191***	-.098	**-.178***	-.041	-.039	-.018	-.101
PEA	-.113	-.077	-.038	-.126	-.117	-.077	-.089	-.118	-.135	-.042	-.012	-.039
	Children *N* = 92						Children *N* = 170					
	CM load	Emotional abuse	Physical abuse	Sexual abuse	Emotional neglect	Physical neglect	CM load	Emotional abuse	Physical abuse	Sexual abuse	Emotional neglect	Physical neglect
AEA	---	---	---	---	---	---	-.023	.095	-.043	.071	-.029	.019
2-AG/1-AG	.147	.152	.057	-.046	**.205*** [b]	.041	.086	-.023	-.062	.034	.079	.124
OEA	.134	.075	.037	-.122	**.268****	.014	-.046	-.037	-.030	.021	-.040	.047
SEA	.028	.001	.018	-.116	.161	-.012	**-.151***	-.143	-.058	-.013	-.104	-.150
PEA	.012	-.034	.003	*-.202*	*.184*	-.068	-.044	-.080	-.096	.075	-.003	.058

Note: * $p < .050$, ** $p < .010$, *** $p < .001$, two-tailed. Underlined *p*-values are significant after correction with false discovery rate (FDR). Bivariate correlations were computed with the maximal number of cases available: [a]$n = 39$; [b]$n = 97$; [c]$n = 88$. Note that no AEA levels were detectable in the hair of children at t_0. Exposure to childhood maltreatment (CM) was assessed with the Childhood Trauma Questionnaire (CTQ, Bader et al., 2009).

Supplementary Table S6 Bivariate Spearman rank correlations *(rs)* between endocannabinoid concentrations in hair and covariates in *N* = 45 children

		Children t_0			
		**2-AG/1-AG**	**OEA**	**SEA**	**PEA**
**Gestational age**	*rs*	*.290*	.174	.015	.104
	p	.054	.253	.922	.496
**Birth weight**	*rs*	.016	-.029	-.184	-.227
	p	.917	.850	.227	.133
**Sex**	*rs*	.083	-.010	-.010	-.080
	p	.587	.946	.946	.603
**Hair weight**	*rs*	*.287*	-.107	**-.407****	**-.369***
	p	.056	.484	.005	.013
**Hair color**	*rs*	.125	.058	.141	-.075
	p	.415	.705	.355	.626

		Children t_2			
		**2-AG/1-AG**	**OEA**	**SEA**	**PEA**
**Age in days**	*rs*	*-.285*	-.113	*-.278*	-.246
	p	.058	.460	.064	.103
**Weight**	*rs*	-.007	.034	-.034	.102
	p	.964	.826	.825	.505
**Sex**	*rs*	.118	.208	.118	-.038
	p	.441	.171	.441	.804
**Serious somatic illness since birth**	*rs*	-.066	.100	.174	.050
	p	.665	.515	.252	.745
**Regular medication since birth**	*rs*	-.022	-.130	-.054	-.175
	p	.884	.395	.726	.251
**Hair weight**	*rs*	-.177	.083	.114	-.057
	p	.245	.588	.456	.711
**Hair color**	*rs*	-.116	.249	-.158	.017
	p	.447	.099	.301	.914

Note: * *p* < .050, ** *p* < .010, *** *p* < .001, two-tailed. Italic *p*-values indicate a trend for significance (p < .100). Underlined *p*-values are significant after correction with false discovery rate (FDR). data: [a] Due to many AEA measures under technical detection limit, there is not sufficient AEA data in children available for analyses.

Supplementary Table S7 Bivariate Spearman rank correlations *(r*s) between endocannabinoid concentrations in hair and covariates in *N* = 63 mothers

		**Mothers t0**				
		**AEA**[a]	**2-AG/1-AG**	**OEA**	**SEA**	**PEA**
**Age**	*rs*	.177	-.073	.064	.065	.046
	p	.496	.569	.616	.615	.718
**Perceived Stress (PSS4)**	*rs*	.322	.056	-.070	-.070	-.003
	p	.208	.663	.585	.585	.983
**Psychiatric diagnose lifetime**	*rs*	-.201	.030	*-.218*	**-.253***	-.200
	p	.439	.815	.086	.045	.116
**Psychotropic medication lifetime**	*rs*	.311	.087	.064	-.053	.031
	p	.224	.497	.616	.683	.809
**Weekly hair washing frequency**	*rs*	-.168	.028	**.282***	.248	.221
	p	.549	.831	.029	.056	.089
**Hair treatment**	*rs*	**.640****	.055	.116	.173	.100
	p	.006	.673	.371	.178	.441
**Hair weight**	*rs*	-	**-.266***	.010	.090	.032
	p	-	.035	.940	.483	.801
**Smoking pregnancy**	*rs*	-.102	.012	.020	-.053	.025
	p	.697	.924	.873	.678	.848

		**Mothers t2**				
		**AEA**[b]	**2-AG/1-AG**	**OEA**	**SEA**	**PEA**
**Age**	*rs*	-.048	-.007	-.061	-.130	-.053
	p	.830	.959	.632	.310	.679
**Perceived stress (PSS14)**[c]	*rs*	.074	.206	.126	-.005	.043
	p	.742	.112	.333	.969	.744
**Psychiatric diagnosis lifetime**[d]	*rs*	.180	.041	**-.288***	*-.220*	*-.250*
	p	.422	.762	.028	.098	.059
**Psychotropic medication intake since birth** [c]	*rs*	.081	-.129	.069	.151	.065
	p	.715	.321	.598	.246	.621
**Weekly hair washing frequency**[c]	*rs*	-.095	.036	**.324***	**.296***	**.373****
	p	.683	.785	.012	.021	.003
**Hair treatment**	*rs*	-.398	-.142	.060	-.313	.055
	p	*.082*	.539	.797	.167	.814
**Sport frequency per week**[c]	*rs*	-.044	.079	**.474****	.146	**.386***
	p	.905	.674	.007	.432	.032
**BMI** (kg/m^2)[c]	*rs*	-.209	.107	-.040	-.054	.028
	p	.362	.421	.763	.686	.836
**Currently Smoking** [c]	*rs*	-.165	-.056	.141	.088	.141

	p	.453	.667	.279	.502	.279
**Current Medication intake** [b]	r_S	-.141	.081	.030	.037	.028
	p	.520	.533	.819	.775	.830

Note: * $p < .050$, ** $p < .010$, *** $p < .001$, two-tailed. Italic *p*-values indicate a trend for significance (p < .100). Underlined *p*-values are significant after correction with false discovery rate (FDR).
data: [a] $N = 17$; [b] $N = 23$
[c] $N = 61$ for 2-AG/1-AG, OEA, SEA & PEA and $N = 21$ for AEA;
[c] $N = 58$ for 2-AG/1-AG, OEA, SEA & PEA and $N = 21$ for AEA;
[c] $N = 31$ for 2-AG/1-AG, OEA, SEA & PEA and $N = 10$ for AEA;
PSS-4/-14 4-/ 14-Item Perceived Stress Scale

Supplementary Statistical Analyses

1. **Group differences in endocannabinoid hair concentrations depending on exposure to childhood maltreatment during pregnancy and one year postpartum**

In accordance with our preceding study (Koenig et al., 2018) and for reasons of completeness we report supplementary analyses on CM-related group differences in eCB and NAE in mothers and children.

The following results can be found in Table S6 and Figure S2 and S3 for the sample of *N*=63 mothers and *N*=45 children with complete hair data at t_0 and t_2 who were included in the longitudinal analyses. Supplementary Table S7 and Figure S4 report the results using the complete available sample at t_0 ($N_m = 150$; $N_c = 92$) and t_2 ($N_m = 148$; $N_c = 170$), respectively.

Maternal hair. Partially confirming our prior results from a bigger study cohort within the "My Childhood – Your Childhood" study (see Koenig et al., 2018), in *N*=63 mothers at t_0 maternal SEA concentrations were significantly lower in CM+ as compared to CM- women. 2-AG/1-AG was descriptively higher in CM+ women as compared to CM- women, but failed to reach significance. OEA and PEA were descriptively lower in CM+ women as compared to CM- women, but failed to reach significance. At 12 months postpartum (t_2) these effects vanished: eCB and NAE hair concentrations did neither differ between mothers with and without a history of CM.

Infant hair. eCB levels in $N = 45$ newborn's hair were analyzed with respect to a maternal history of CM. At t_0, in newborns with CM+ mothers, OEA hair concentrations were significantly higher than in newborns without maternal CM, whereas 2-AG/1-AG hair concentrations were descriptively higher in newborns with CM+ mothers than in newborns of CM- mothers. For SEA and PEA no statistical differences were found regarding maternal CM status. At 12 months postpartum (t_2) these effects vanished: eCB and NAE hair concentrations did neither differ between children with and without a maternal history of CM

Supplementary Table S8. Wilcoxon rank-sum test on differences in endocannabinoid concentrations in N = 63 mothers and N = 45 children with complete data at both points of measurement depending on maternal CM load

t_0 hair samples representing the last trimester of pregnancy

	Mothers				Children			
	**CM+ (n = 37)**	**CM- (n = 26)**			**CM+ (n = 21)**	**CM- (n = 24)**		
	**Med (IQR)**	**Med (IQR)**	***Z***	***p***	**Med (IQR)**	**Med (IQR)**	***Z***	***p***
**2-AG/1-AG**	21.77 (24.50)	14.04 (19.36)	-1.480	.139	74.30 (154.70)	60.89 (120.24)	-1.206	.228
**OEA**	1805.63 (2616.89)	1883.03 (3265.46	-.824	.410	91.32 (97.36)	57.59 (67.60)	-1.684	***.092***
**SEA**	677.23 (559.81)	1297.11 (1602.96)	-2.862	**<.004****	474.20 (610.03)	378.31(426.23)	-.978	.328
**PEA**	1495.13 (2575.54	2504.12 (4424.66)	-1.550	.121	153.21 (205.88)	132.18 (180.13)	-.466	.641

t_2 hair samples representing 10-12 months postpartum

	Mothers				Children			
	**CM+ (n = 37)**	**CM- (n = 26)**			**CM+ (n = 21)**	**CM- (n = 24)**		
	**Med (IQR)**	**Med (IQR)**	***Z***	***p***	**Med (IQR)**	**Med (IQR)**	***Z***	***p***
**2-AG/1-AG**	30.81 (37.37)	24.85 (21.09)	-1.061	.289	10.69 (14.09)	12.29 (19.73)	-.728	.467
**OEA**	1002.38 (2897.88)	1075.10 (3277.85)	-.768	.443	407.65 (416.31)	504.08 (367.16)	-1.069	.285
**SEA**	479.25 (753.50)	514.65 (899.65)	-1.384	.167	553. 73 (288.82)	597.46 (420.17)	-1.229	.219
**PEA**	1535.38 (3526.70)	1334.58 (4127.04)	-.600	.548	508.18 (304.40)	499.70 (234.09)	-.091	.927

Note: * p < .050, ** p < .010, *** p < .001; Two-sided Wilcoxon rank-sum Test. Italic p values indicate a trend for significance (p < .100). CM + vs. CM-; Underlined p-values are significant after correction with false discovery rate (FDR). According to the mild cut-off criterion of the CTQ (Bernstein & Fink, 1998) women without any CM experiences were classified as CM− and those with at least mild CM experiences in at least one CTQ subscale were categorized as CM+. Wilcoxon tests were computed using all available samples of mother-child-dyads with hair samples collected at t_0 and t_2. Note that at t_0, AEA was analyzed in a subsample of mothers only. As many of the AEA measures were below the technical detection limit, AEA had to be excluded from Wilcoxon rank-sum tests as there was no sufficient data. For analysis and interpretation *Med* and *IQR* are used exclusively. CM Childhood Maltreatment; AEA anandamide, 2-AG/1-AG 2/1-arachidonoylglycerol; OEA N-oleoylethanolamide; SEA N-stearoylethanolamide; PEA N-palmitoylethanolamide.

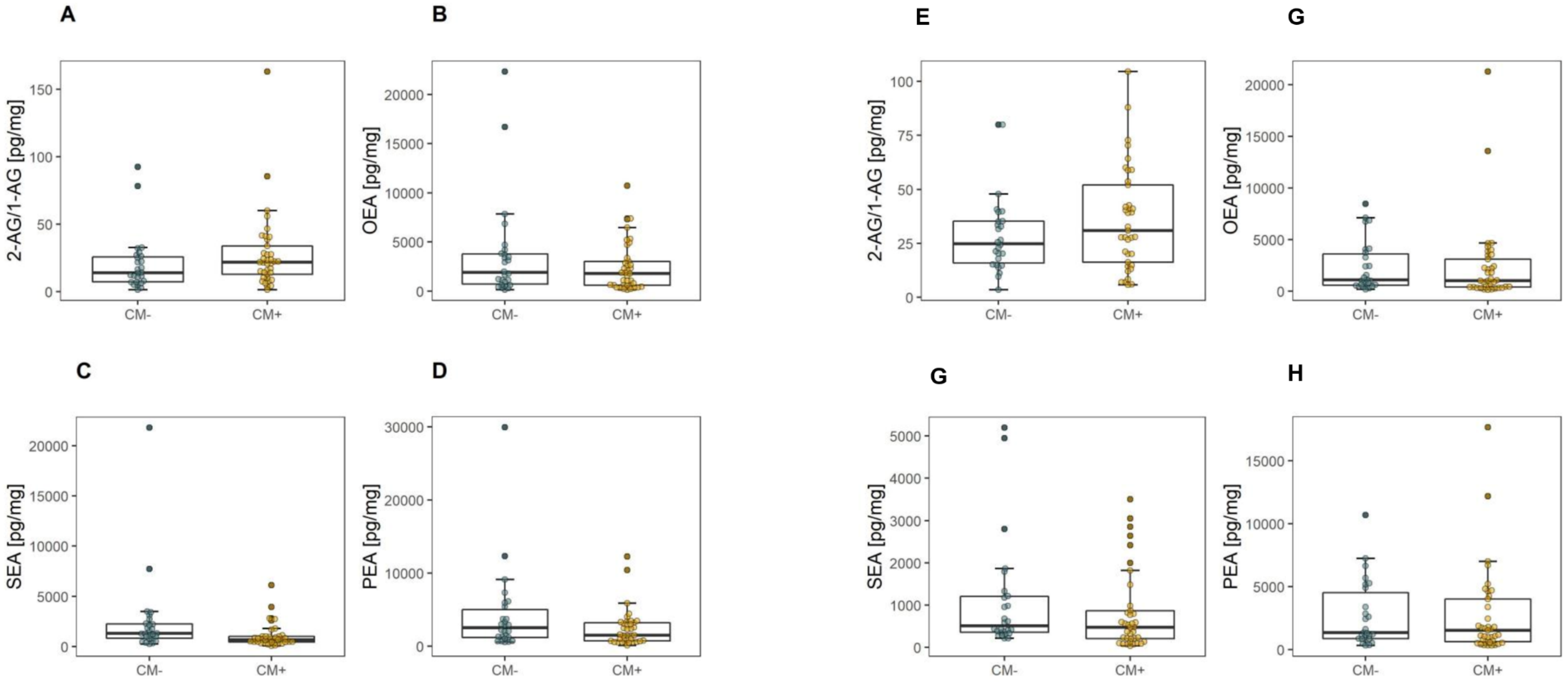

Supplementary Figure S3. Comparison of *N* = 63mothers with (CM+) and without childhood maltreatment (CM-) experiences in late pregnancy (t_0, A-D) and one year after birth (t_2, E-H) regarding their eCB and NAE hair concentrations represented by boxplots with overlying bee-swarms. Endocannabinoids are presented as raw data in pg/mg. CM+, women with CM experiences; CM-, women without CM experiences; 2-AG/1-AG 2/1-arachidonoylglycerol; OEA N-oleoylethanolamide; SEA N-stearoylethanolamide; PEA N-palmitoylethanolamide.

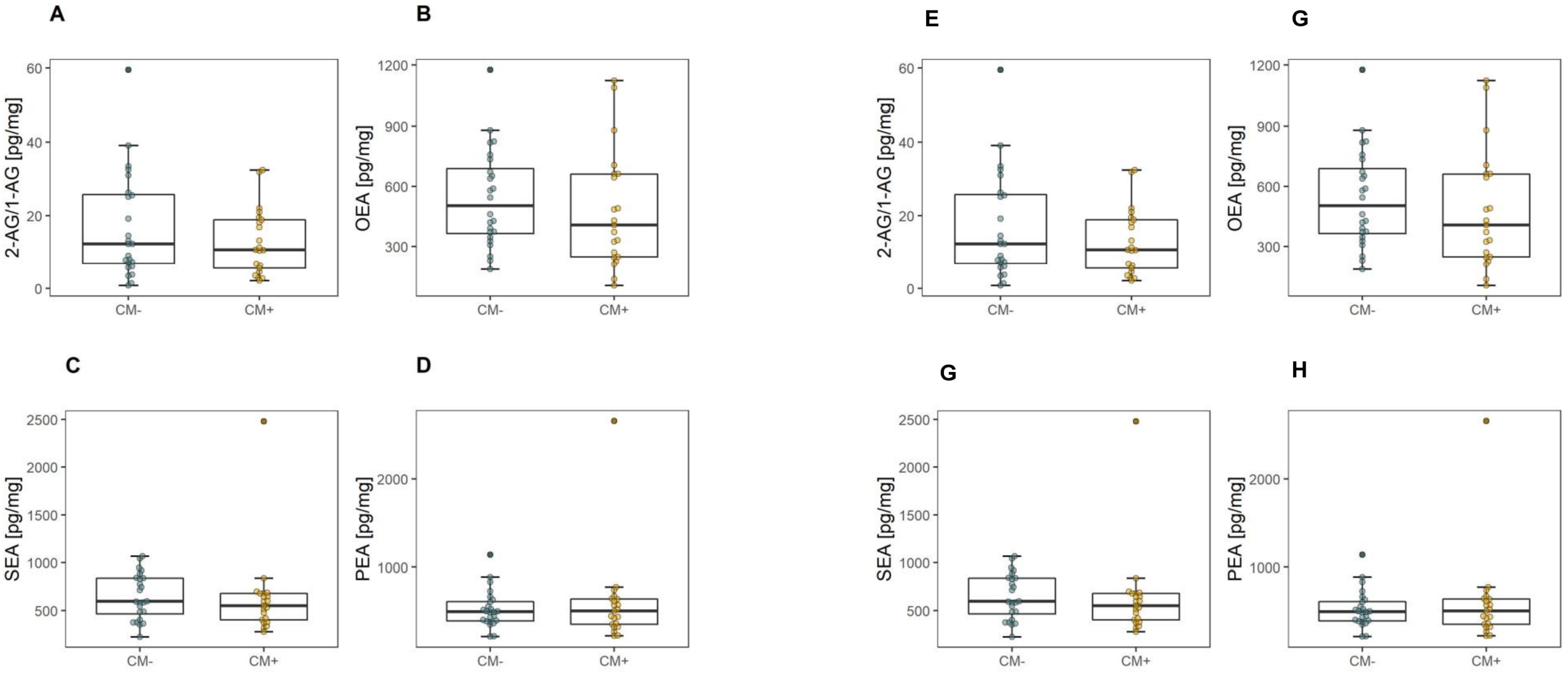

Supplementary Figure S4. Comparison of $N = 45$ children with (CM+) and without mothers exposed to childhood maltreatment (CM-) experiences in late pregnancy (t_0, A-D) and one year after birth (t_2, E-H) regarding their eCB and NAE hair concentrations represented by boxplots with overlying bee-swarms. Endocannabinoids are presented as raw data in pg/mg. CM+, women with CM experiences; CM-, women without CM experiences; 2-AG/1-AG 2/1-arachidonoylglycerol; OEA N-oleoylethanolamide; SEA N-stearoylethanolamide; PEA N-palmitoylethanolamide.

Supplementary Table S9. Wilcoxon rank-sum test on differences in endocannabinoid concentrations in mothers and children depending on maternal CM load for the complete available sample

	**t_0 hair samples representing the last trimester of pregnancy**									
	**Mothers (*N* = 150)***					**Children (*N* =92)***				
	**CM+ (n = 76)**	**CM- (n = 74)**				**CM+ (n = 37)**	**CM- (n = 55)**			
	**Med (IQR)**	**Med (IQR)**	***W***	***p***	***r***	**Med (IQR)**	**Med (IQR)**	***W***	***p***	***r***
**2-AG/1-AG**	21.86 (25.82)	15.14 (15.50)	2097.50	**.007****	-.22	100.05 (114.00)	62.72 (95.63)	770.00	**.049***	-.021
**OEA**	1685.82 (3110.54)	1687.52 (3474.40)	291.00	.695	-.32	91.32 (100.63)	61.66 (90.89)	785.00	*.064*	-.19
**SEA**	623.94 (791.21)	1297.11 (1431.19)	3903.50	**<.000*****	-.33	562.89 (754.00)	497.53 (595.41)	898.00	.343	-.99
**PEA**	1529.50 (2734.35)	2103.40 (3189.15)	3184	.163	-.12	162.38 (218.21)	139.76 (220.18)	970.50	.711	-.04

	**t_0 hair samples representing 10-12 months postpartum**									
	**Mothers (*N* = 148)**					**Children (*N* = 170)**				
	**CM+ (n = 74)**	**CM- (n = 74)**				**CM+ (n = 75)**	**CM- (n = 94)**			
	**Med (IQR)**	**Med (IQR)**	***W***	***p***	***r***	**Med (IQR)**	**Med (IQR)**	***W***	***p***	***r***
**AEA**	0.46 (0.28)	0.39 (0.26)	1032.00	.254	-.94	0.25 (0.17)	0.28 (0.14)	954.00	.913	-.01
**2-AG/1-AG**	26.75 (29.06)	22.10 (50.59)	2545.50	.462	-0.06	33.43 (50.22)	27.54 (35.90)	3131.50	.214	-.10
**OEA**	1108.46 (2520.21)	1096.89 (2783.47)	2857.00	.650	-0.04	614.81 (534.86)	657.12 (376.28)	3714.50	.550	-.05
**SEA**	397.64 (650.52)	344.24 (625.55)	2665.00	.781	-0.02	443.70 (325.19)	485.90 (416.06)	3972.00	.157	-.11
**PEA**	1509.36 (2784.92)	1343.32 (3040.59)	2707.50	.908	-0.01	620.11 (429.01)	608.65 (30.41)	3522.50	.995	-.001

Note: * $p < .050$, ** $p < .010$, *** $p < .001$; Two-sided Wilcoxon rank-sum Test. Italic *p* values indicate a trend for significance (p < .100). CM + vs. CM-; Underlined *p*-values are significant after correction with false discovery rate (FDR). *r* effect size measure

According to the mild cut-off criterion of the CTQ (Bernstein & Fink, 1998) women without any CM experiences were classified as CM− and those with at least mild CM experiences in at least one CTQ subscale were categorized as CM+. Wilcoxon tests were computed using all available samples of mother-child-dyads with hair samples collected at t_0 and t_2. Note that at t_0, AEA was analyzed in a subsample of mothers only. As many of the AEA measures were below the technical detection limit, AEA had to be excluded from Wilcoxon rank-sum tests as there was no sufficient data for mothers-child-dyads. For analysis and interpretation *Med* and *IQR* are used exclusively.
Abbreviations: CM Childhood Maltreatment; AEA anandamide, 2-AG/1-AG 2/1-arachidonoylglycerol; OEA N-oleoylethanolamide; SEA N-stearoylethanolamide; PEA N-palmitoylethanolamide.
* Results based on the same study cohort were previously published in Koenig et al., 2018, and replicated within the current study, however, using a different statistical approach

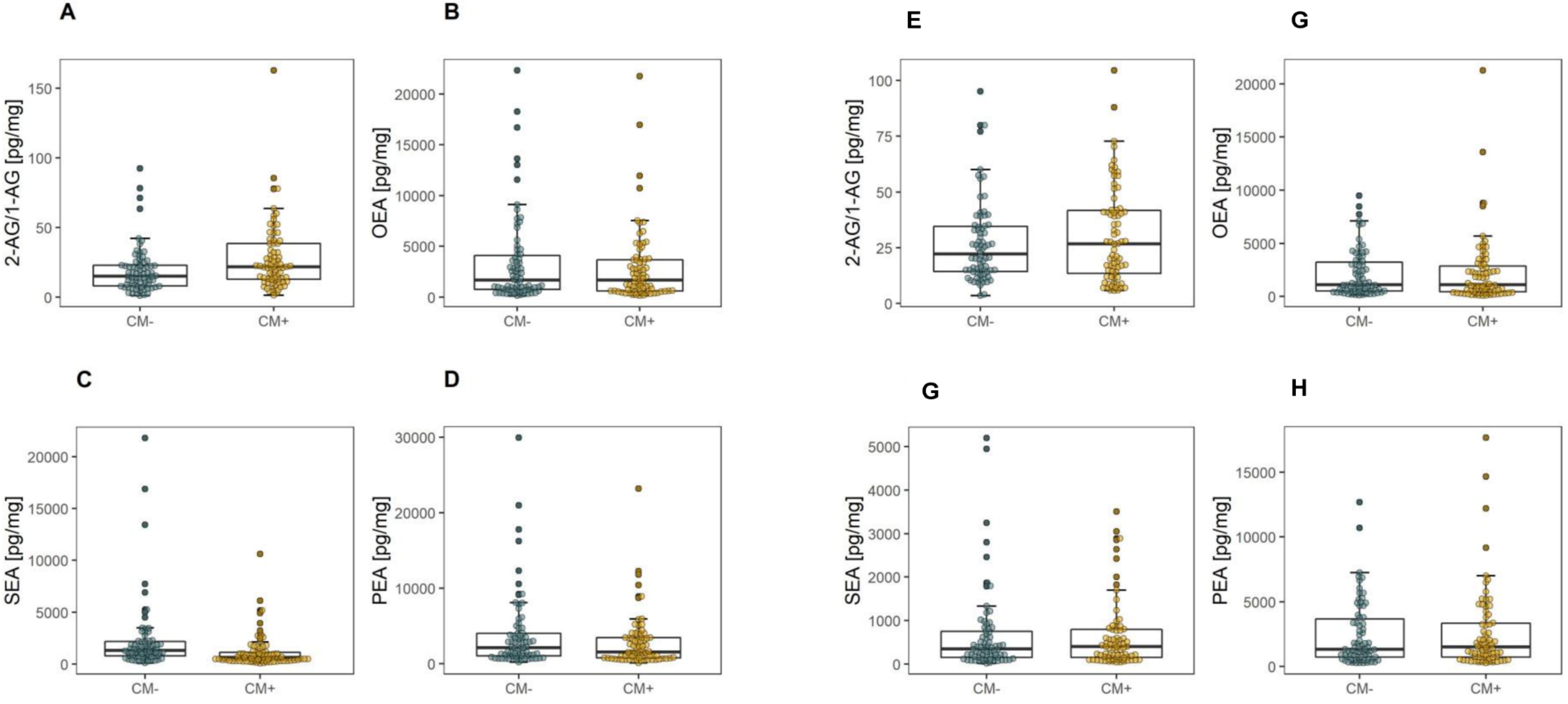

Supplementary Figure S5. Comparison of mothers with (CM+) and without childhood maltreatment (CM-) experiences in late pregnancy (t_0, A-D; $N = 150$) and one year after birth (t_2, E-H; $N = 148$) regarding their eCB and NAE hair concentrations represented by boxplots with overlying bee-swarms. Endocannabinoids are presented as raw data in pg/mg. CM+, women with CM experiences; CM-, women without CM experiences; 2-AG/1-AG 2/1-arachidonoylglycerol; OEA N-oleoylethanolamide; SEA N-stearoylethanolamide; PEA N-palmitoylethanolamide.

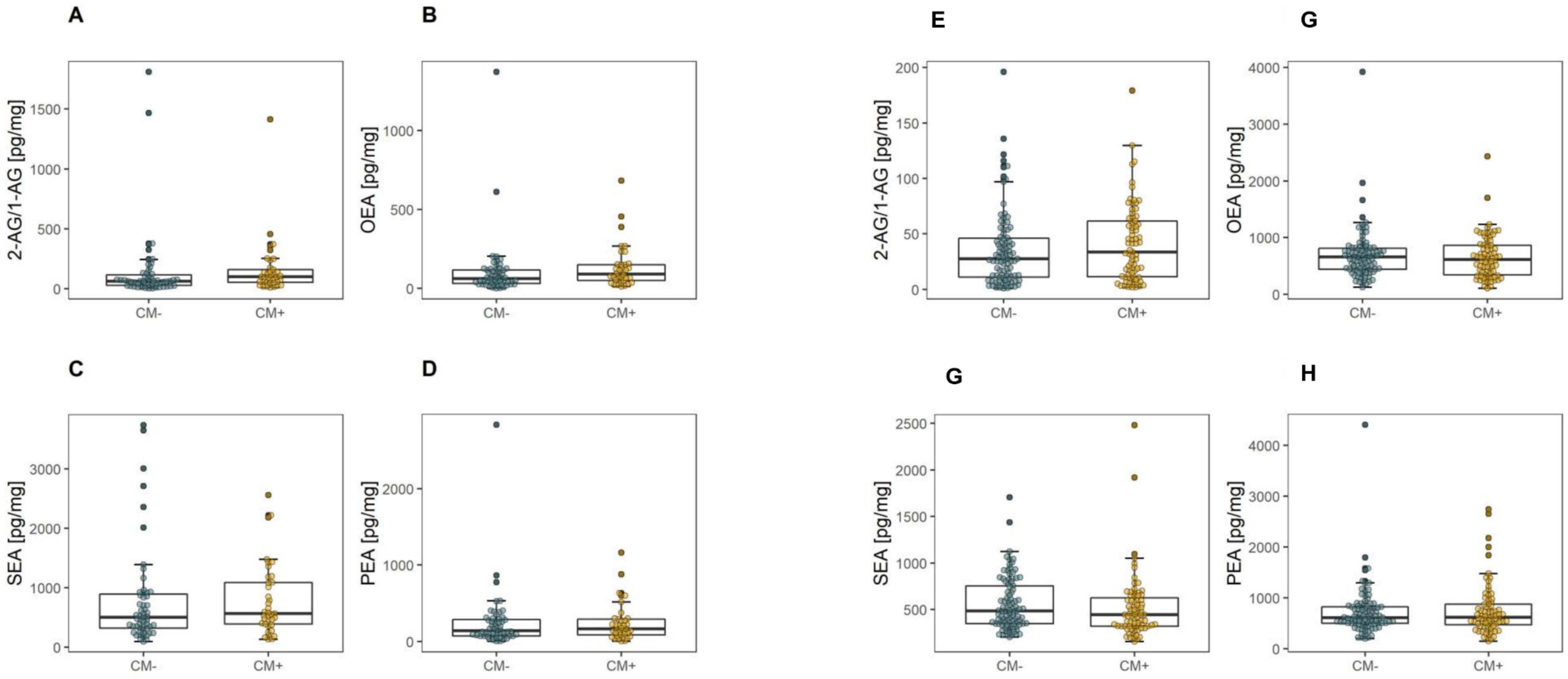

Supplementary Figure S6. Comparison of children with mothers with (CM+) and without exposure to childhood maltreatment (CM-) in late pregnancy (t_0, A-D; n = 92) and one year after birth (t_2, E-H; n = 170) regarding their eCB and NAE hair concentrations represented by boxplots with overlying bee-swarms. Endocannabinoids are presented as raw data in pg/mg. CM+, women with CM experiences; CM-, women without CM experiences; 2-AG/1-AG 2/1-arachidonoylglycerol; OEA N-oleoylethanolamide; SEA N-stearoylethanolamide; PEA N-palmitoylethanolamide.

2. Longitudinal analyses of eCB and NAE in mothers and children with complete data at both points of measurement

Supplementary Table S10 Results of robust linear mixed effect models for endocannabinoid concentrations in mothers (*N* = 63)

Outcome	Predictor	*b*	95% CI (*b*)	*β*	η^2_p [95% CI] #	*t*	*p*
2-AG/1-AG	Intercept	18.44	6.15 – 30.72	-0.34	0.07 [0.01 – 0.17]	2.94	**<.001*** **
	Time	11.50	-5.87 – 28.88	0.38	0.01 [0.00 – 0.08]	1.30	**.004** **
	CM	0.06	-0.24 – 0.37	0.04	0.00 [0.00 – 0.04]	0.41	.684
	Time x CM	-0.06	-0.49 – 0.38	-0.03	0.00 [0.00 – 0.03]	-0.26	.793
	Model statistics: conditional R^2= .068, marginal R^2 = .068, σ_{ri} = 17.59; RMSE = 24.207						
OEA	Intercept	2506.42	1092.50 – 3920.34	-0.13	0.07 [0.01 – 0.17]	2.94	.070
	Time	212.34	-1787.25 – 2211.93	-0.10	0.01 [0.00 – 0.08]	1.30	.314
	CM	-8.36	-43.86 – 27.13	-0.03	0.00 [0.00 – 0.04]	0.41	.644
	Time x CM	-15.75	-65.95 – 34.44	-0.06	0.00 [0.00 – 0.03]	-0.26	.538
	Model statistics: conditional R^2= .025, marginal R^2 = .025, σ_{ri} = 2024; RMSE = 684.48						
PEA	Intercept	3045.72	1577.58 – 4513.87	-0.16	0.07 [0.01 – 0.17]	2.94	**.028***
	Time	188.17	-1888.09 – 2264.44	-0.04	0.01 [0.00 – 0.08]	1.30	.688
	CM	-18.53	-55.39 – 18.32	-0.07	0.00 [0.00 – 0.04]	0.41	.324
	Time x CM	-9.22	-61.34 – 42.90	-0.03	0.00 [0.00 – 0.03]	-0.26	.729
	Model statistics: conditional R^2= .027, marginal R^2 = .027, σ_{ri} = 2101; RMSE = 3825.75						
SEA	Intercept	1703.25	1143.78 – 2262.71	-0.09	0.23 [0.11 – 0.35]	5.97	**.048***
	Time	-521.73	-1312.94 – 269.47	-0.16	0.03 [0.00 – 0.15]	-1.29	**.013***
	CM	-16.16	-30.20 – -2.11	-0.10	0.04 [0.00 – 0.13]	-2.26	**.024***
	Time x CM	4.29	-15.58 – 24.15	0.03	0.00 [0.00 – 0.08]	0.42	.672

Model statistics: conditional R^2= .105, marginal R^2 = .105, σ_{ri} = 800.8; RMSE = 2206.55

Note. * p < .050, ** p < .010, *** p < .001, two-tailed. All models include random intercepts to consider repeated measures within individuals (σ_{ri} standard deviation of random intercepts). Coefficients of determination (*conditional* R^2) present the variance explained by the total model (fixed and random effects) and *marginal* R^2 the variance explained by fixed effects only. σ_{ri} presents the standard deviation of random intercepts across all subjects; RMSE presents the absolute model-to-data-fit by estimating the unexplained variance (quantified deviation of the estimated from the predicted values). Overall model tests cannot be calculated for robust linear mixed effects models. Exposure to childhood maltreatment (CM) was assessed with the sum score of the Childhood Trauma Questionnaire (CTQ, Bader et al., 2009).

Supplementary Table S11 Results of robust linear mixed effect models for endocannabinoid concentrations in children (N = 45)

Outcome	Predictor	b	95% CI (b)	β	η^2_p [95% CI]#	t	p
2-AG/1-AG	Intercept	70.12	45.86 – 94.37	-0.02	0.27 [0.13 – 0.41]	5.67	.446
	Time	-52.08	-86.38 – -17.78	-0.34	0.17 [0.02 – 0.37]	-2.98	**<.001*** **
	CM load	0.03	-0.58 – 0.65	0.00	0.004 [0.00 – 0.03]	0.11	.913
	Time x CM	-0.12	-0.99 – 0.75	-0.01	0.00 [0.00 – 0.09]	-0.27	.791
	Model statistics: conditional R^2= .424, marginal R^2 = .424, σ_{ri} = 33.03; RMSE = 159.99						
OEA	Intercept	51.28	-70.63 – 173.19	-0.74	0.01 [0.00 – 0.80]	0.82	**<.001*** **
	Time	482.70	310.29 – 655.10	1.31	0.41 [0.19 – 0.58]	5.49	**<.001*** **
	CM load	1.27	-1.82 – 4.36	0.07	0.01 [0.00 – 0.09]	0.81	.420
	Time x CM	-2.90	-7.27 – 1.47	-0.16	0.04 [0.00 - 0.20]	-1.30	.193
	Model statistics: conditional R^2= .571, marginal R^2 = .571, σ_{ri} = 166; RMSE = 203.24						
PEA	Intercept	141.18	25.01 – 257.35	-0.59	0.06 [0.00 – 0.18]	2.38	**<.001*** **
	Time	414.37	250.08 – 578.65	0.94	0.36 [0.14 – 0.54]	4.94	**<.001*** **
	CM load	1.07	-1.87 – 4.02	0.05	0.01 [0.00 – 0.08]	0.71	.475
	Time x CM	-2.50	-6.67 – 1.66	-0.12	0.00 [0.00 – 0.19]	-1.18	.239

	Model statistics: conditional R^2= .519, marginal R^2 = .519, σ_{ri} = 158.2; RSME = 296.5						
SEA	Intercept	407.92	208.36 – 607.49	-0.31	0.17 [0.04 – 0.31]	4.01	**.003****
	Time	229.53	-52.70 – 511.75	0.29	0.06 [0.00 – 0.23]	1.59	**.048***
	CM load	2.17	-2.88 – 7.23	0.09	0.01 [0.00 – 0.09]	0.84	.400
	Time x CM	-3.15	-10.30 – 4.01	-0.13	0.02 [0.00 – 0.16]	-0.86	.388
	Model statistics: conditional R^2= .053, marginal R^2 = .053, σ_{ri} = 271.7; RMSE = 394.73						

Note. * $p < .050$, ** $p < .010$, *** $p < .001$, two-tailed. All models include random intercepts to consider repeated measures within individuals (σ_{ri} standard deviation of random intercepts). Coefficients of determination (*conditional* R^2) present the variance explained by the total model (fixed and random effects) and *marginal* R^2 the variance explained by fixed effects only. σ_{ri} presents the standard deviation of random intercepts across all subjects; RMSE presents the absolute model-to-data-fit by estimating the unexplained variance (quantified deviation of the estimated from the predicted values). Overall model tests cannot be calculated for robust linear mixed effects models. Exposure to childhood maltreatment (CM) was assessed with the sum score of the Childhood Trauma Questionnaire (CTQ; Bader et al., 2009).

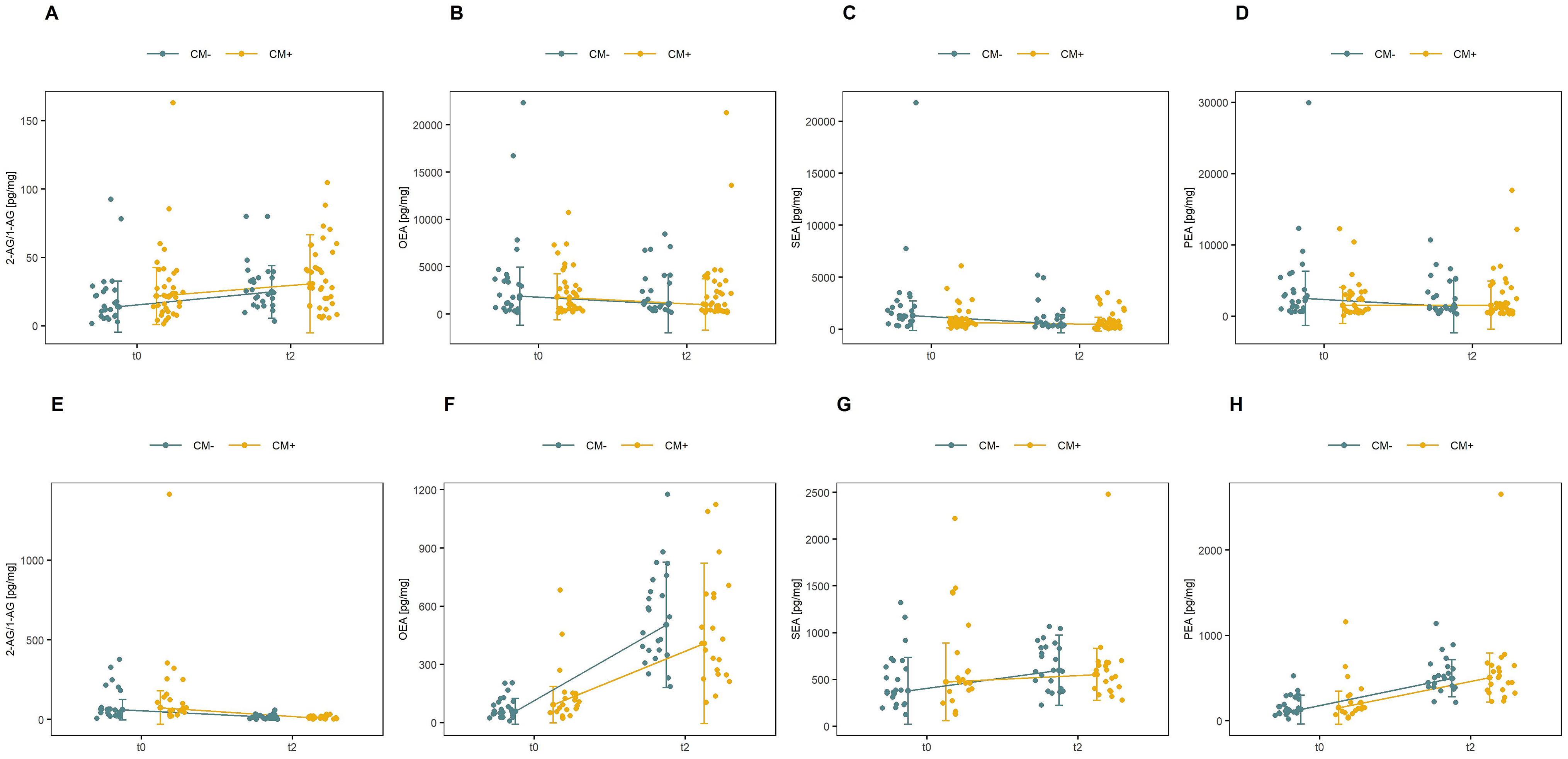

Figure S7. Course of endocannabinoids depending on maternal CM. Endocannabinoid (eCB) and N-acylethanolamines (NAE) hair concentrations (pg/mg) in mothers (A-D; *N* = 63) and their children (E-H; *N* = 45) with lower (CM-) and higher childhood maltreatment (CM+) load representing last trimester of pregnancy and 12 months postpartum. t0 hair sampled shortly after birth, representing the last trimester of pregnancy; t2 hair sampled 12 months postpartum, representing 10 to 12 months postpartum. 2-AG/1-AG 2-arachidonoylglycerol, SEA stearoylethanolamide, OEA oleoylethanolamide, PEA palmitoylethanolamide.

References

Bader, K., Hänny, C., Schäfer, V., Neuckel, A., & Kuhl, C. (2009). Childhood Trauma Questionnaire - Psychometrische Eigenschaften einer deutschsprachigen Version. *Zeitschrift für Klinische Psychologie und Psychotherapie, 38*(4), 223–230. https://doi:10.1026/1616-3443.38.4.223

Bernstein, D. P., & Fink, L. (1998). Childhood Trauma Questionnaire: A retrospective self-report. San Antonio, TX: The Psychological Corporation.

de Kruijff, I., Noppe, G., Kieviet, N., Choenni, V., Lambregtse-van den Berg, M. P., Begijn, D. G., ... & van den Akker, E. L. (2020). LC-MS/MS-based reference intervals for hair cortisol in healthy children. *Psychoneuroendocrinology*, *112*, 104539. https://doi.org/10.1016/j.psyneuen.2019.104539

Gao, W., Walther, A., Wekenborg, M., Penz, M., & Kirschbaum, C. (2020). Determination of endocannabinoids and N-acylethanolamines in human hair with LC-MS/MS and their relation to symptoms of depression, burnout, and anxiety. *Talanta*, *217*, 121006. https://doi.org/10.1016/j.talanta.2020.121006

Gareri, J., & Koren, G. (2010). Prenatal hair development: Implications for drug exposure determination. *Forensic Science International, 196*(1–3), 27–31. https://doi.org/10.1016/j.forsciint.2009.12.024

Koenig, A. M., Gao, W., Umlauft, M., Schury, K., Reister, F., Kirschbaum, C., ... & Kolassa, I. T. (2018). Altered hair endocannabinoid levels in mothers with childhood maltreatment and their newborns. *Biological Psychology, 135*, 93-101. https://doi.org/10.1016/j.biopsycho.2018.03.006

Krumbholz, A., Anielski, P., Reisch, N., Schelling, G., & Thieme, D. (2013). Diagnostic value of concentration profiles of glucocorticosteroids and endocannabinoids in hair. *Therapeutic Drug Monitoring, 35*(5), 600–7. https://doi.org/10.1097/ftd.0b013e3182953e43

Pecoraro, V., Astore, I., Barman, J., & Araujo, C. I. (1964). The normal trichogram in the child before the age of puberty. Journal of Investigative Dermatology, 42(6), 427-430. https://doi.org/10.1038/jid.1964.92

Vaughn, L. K., Denning, G., Stuhr, K. L., de Wit, H., Hill, M. N., & Hillard, C. J. (2010). Endocannabinoid signalling: has it got rhythm?. *British Journal of Pharmacology, 160*(3), 530-543. https://doi.org/10.1111/j.1476-5381.2010.00790.x

Wennig, R. (2000). Potential problems with the interpretation of hair analysis results. *Forensic Science International, 107*, 1–3. https://doi.org/10.1016/s0379-0738(99)00146-2
